# Integrating Mechanics and Bioactivity: A Detailed Assessment of Elasticity and Viscoelasticity at Different Scales in 2D Biofunctionalized PEGDA Hydrogels for Targeted Bone Regeneration

**DOI:** 10.1021/acsami.4c10755

**Published:** 2024-07-23

**Authors:** Cristina López-Serrano, Yeva Côté-Paradis, Birgit Habenstein, Antoine Loquet, Cédric Le Coz, Jean Ruel, Gaétan Laroche, Marie-Christine Durrieu

**Affiliations:** † Univ. Bordeaux, CNRS, Bordeaux INP, CBMN, UMR 5248, Pessac 33600, France; ‡ Laboratoire d’Ingénierie de Surface, Centre de Recherche sur les Matériaux Avancés, Département de Génie des Mines, de la Métallurgie et des Matériaux, Université Laval, Québec, QC G1 V 0A6, Canada; § Axe médecine régénératrice, Centre de Recherche du Centre Hospitalier Universitaire de Québec, Hôpital St-François d’Assise, Québec, QC G1L 3L5, Canada; ∥ Univ. Bordeaux, CNRS, INSERM, IECB, US1, UAR 3033, F-33600 Pessac, France; ⊥ Univ. Bordeaux, CNRS, Bordeaux INP, LCPO, UMR 5629, F-33600 Pessac, France; # Département de Génie Mécanique, Université Laval, Québec, QC G1V 0A6, Canada

**Keywords:** hydrogel, mechanical properties, mesenchymal stem cell, viscoelasticity, osteogenic differentiation

## Abstract

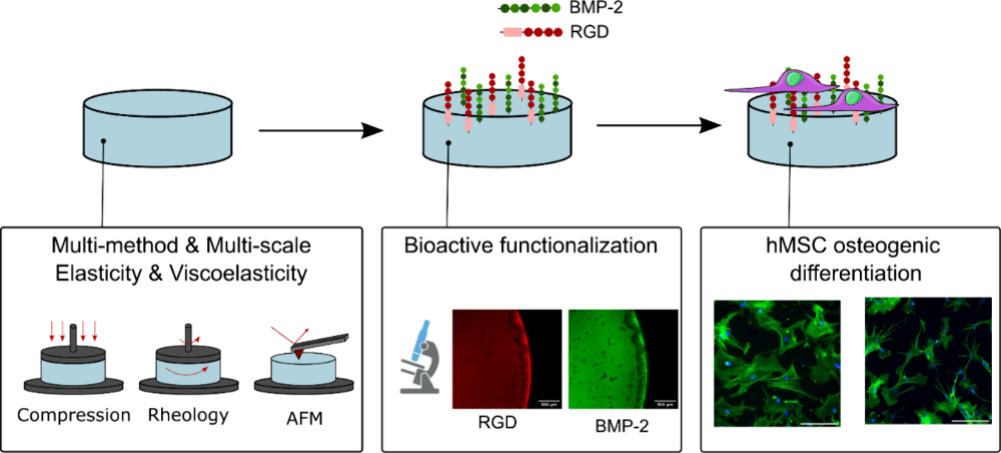

Methods for promoting and controlling the differentiation of human mesenchymal stem cells (hMSCs) in vitro before in vivo transplantation are crucial for the advancement of tissue engineering and regenerative medicine. In this study, we developed poly(ethylene glycol) diacrylate (PEGDA) hydrogels with tunable mechanical properties, including elasticity and viscoelasticity, coupled with bioactivity achieved through the immobilization of a mixture of RGD and a mimetic peptide of the BMP-2 protein. Despite the key relevance of hydrogel mechanical properties for cell culture, a standard for its characterization has not been proposed, and comparisons between studies are challenging due to the different techniques employed. Here, a comprehensive approach was employed to characterize the elasticity and viscoelasticity of these hydrogels, integrating compression testing, rheology, and atomic force microscopy (AFM) microindentation. Distinct mechanical behaviors were observed across different PEGDA compositions, and some consistent trends across multiple techniques were identified. Using a photoactivated cross-linker, we controlled the functionalization density independently of the mechanical properties. X-ray photoelectrin spectroscopy and fluorescence microscopy were employed to evaluate the functionalization density of the materials before the culturing of hMSCs on them. The cells cultured on all functionalized hydrogels expressed an early osteoblast marker (Runx2) after 2 weeks, even in the absence of a differentiation-inducing medium compared to our controls. Additionally, after only 1 week of culture with osteogenic differentiation medium, cells showed accelerated differentiation, with clear morphological differences observed among cells in the different conditions. Notably, cells on stiff but stress-relaxing hydrogels exhibited an overexpression of the osteocyte marker E11. This suggests that the combination of the functionalization procedure with the mechanical properties of the hydrogel provides a potent approach to promoting the osteogenic differentiation of hMSCs.

## Introduction

Achieving improved bone regeneration involves mimicking the cell’s native microenvironment, the stem cell niche. To this end, it is important to improve our understanding of the interactions of mesenchymal stem cells (MSCs) with their environment and how they might affect cell survival, proliferation, and differentiation. In the human body, cells undergo complex multifactorial processes that cannot be replicated in a traditional cell culture in a polystyrene culture dish. The use of tailored scaffolds represents a more advanced step toward in vitro replication of the in vivo cell niches.

Many aspects of biomaterials concerning their effect on cell behavior have been investigated, such as the presence of bioactive molecules and their geometrical organization,^1^ surface topography,^2^ mechanical properties,^3,4^ surface chemistry, or application of external forces. It has been extensively demonstrated over the past few decades that matrix mechanical properties have a direct effect on MSC function, including their differentiation behavior. The biomaterials community has shown a growing interest in the use of hydrogels as cell culture substrates since they can be used as extracellular matrix (ECM) replicates, thanks to their tunable mechanical and chemical properties.^5^ Tissues in the human body span a wide range of mechanical properties.^3^ Most biological tissues can also be considered as composite materials, with hierarchical structures and many exhibiting anisotropy.^3^ The elastic response of these tissues is also generally nonlinear,^6^ as is the case in many synthetic polymer networks, and most of them display viscoelasticity.^7^ In addition, cellular mechanotransduction occurs as a result of the interaction of cells with the surface of biomaterials, and therefore, it becomes important to assess whether the evaluation of the mechanical properties is relevant at the cellular scale. This complexity of the tissues of interest calls for multivariable analysis of the mechanical properties of the hydrogels used for biological applications. However, there is no standard for the mechanical evaluation of soft biomaterials such as hydrogels. There is still an open question in the field regarding which technique is the best to evaluate the mechanical properties of cell culture scaffolds. Published articles use different measurement methods and parameters, which hinder meaningful comparisons across studies. The matter becomes more complicated when viscoelasticity and not just the elastic modulus, is characterized.

Specifically for MSCs, the importance of the substrate mechanical properties on their differentiation toward different lineages has been proven. Many studies have focused on characterizing the effect of hydrogel stiffness, generally reported as Young’s modulus (E) or storage modulus (*G*′), on MSC differentiation. Generally, publications have found osteogenic differentiation in matrices with elastic moduli from 30 to 50 kPa.^8,9^ Interestingly, in some of these cases, the stiffest hydrogels tested were not the ones for which the best osteogenic differentiation was observed, suggesting that there is an optimal range of elasticity in which cells preferentially differentiate toward the osteogenic lineage. However, other authors have found high osteogenic differentiation in softer matrices or very stiff ones up to 190 kPa in modulus.^10^ This might lead to think that the action of substrate stiffness interacts with other parameters such as surface functionalization^11^ or cell density^12^ that can override or act synergistically with each other. In addition, other properties such as viscoelasticity or plasticity contribute to the mechanical behavior of materials and it has been pointed out that they could be acting as hidden variables on prior studies within the biomaterials field.^4^

Recently, particular attention has been paid to viscoelastic materials. Viscoelasticity describes the time-dependent mechanical response to deformation and, as cells interact with ECMs via dynamic processes that occur over a variety of time scales, cellular activity can be affected by viscoelasticity.^4^ Cameron et al.^13^ fabricated polyacrylamide hydrogels with identical elastic moduli but varying viscoelastic properties. Their results indicated that MSCs exhibited greater spreading and showed enhanced osteogenic differentiation on substrates with higher viscoelasticity. Other authors have shown that high viscoelasticity is able to promote osteogenic differentiation of MSCs even with substrates of nonoptimal rigidity,^14^ indicating that there is an interplay between the effects of elasticity and viscoelasticity on cells. However, the optimal viscoelastic parameters for osteogenic differentiation of MSCs remain unclear due to the novelty of the research and the differences in the characterization techniques used to quantify viscoelasticity. Despite a variety of materials for biomedical applications already existing in the literature, few publications highlight the scale at which these mechanical properties affect cell fate. This work provides a comprehensive study of the effects of hydrogels’ mechanical properties at different scales on cell fate.

In addition, the effect of mechanical properties can also vary depending on the presence and distribution of bioactive factors on the material,^9,11,15^ further complicating the comparison of different studies. Synthetic polymers, such as polyacrylamide or poly(ethylene glycol) (PEG), are widely used to fabricate scaffolds for tissue engineering, but they are generally not adapted for cell attachment and require conjugation with bioactive ligands. The use of synthetic biomimetic peptides that contain the amino acid sequences of interest is becoming more and more frequent, as they are easier and cheaper to synthesize than full-length proteins and they grant better control over the binding sites to both the material and cell receptors. A common functionalization strategy involves the use of peptides containing integrin-binding domains, such as the triamino acid sequence arginine-glycine-aspartic acid (RGD), which is present in proteins such as collagen or fibronectin.^16^ Aside from promoting cell adhesion, it also has been shown that the incorporation of RGD into a hydrogel can enhance the osteogenesis of MSCs.^17^ Another important family of molecules used for hydrogel biofunctionalization is differentiation-inducing peptides, which consist of peptide sequences that affect stem cell fate and guide their differentiation. Bone morphogenetic proteins, BMPs, are a family of growth factors that are present during bone formation and regeneration in the human body.^18^ The most often employed BMPs in clinical practice are BMP-2 and BMP-7, although their use is limited by the potential of ectopic bone formation.^18^ For this reason, peptides that contain selected regions of interest from the proteins are used.^9,14,17,19−21^ BMP-2 mimetic peptides are generally derived from either the wrist or the knuckle epitopes of the full protein, which are recognized respectively by BMP receptors (BMPR) type I and II.^22^ In particular for the knuckle epitope, a sequence corresponding to the residues 73–92 has been used in multiple studies.^9,21^ The combined presence of adhesive and osteogenic factors is a promising strategy, as there is a powerful synergistic effect between integrins and growth factor signaling, specifically with the combined presence of RGD and BMP-2 peptides.^17,23^ The majority of works highlighted in the literature demonstrate an accelerated differentiation of MSCs into osteoblasts by altering either the bioactivity of materials (by varying immobilized biomolecules, their distribution, and density)^11,15,19^ or their mechanical properties (by modifying their elasticity or even their viscoelasticity).^8,14,24,25^ Few studies have focused on simultaneously modifying both the bioactivity and mechanical properties within the same material. To unequivocally elucidate the effects of each property on the behavior and differentiation of stem cells, the development of materials in which they can be tuned independently of each other is needed.

In this context, this study aims to provide a comprehensive mechanical characterization, in terms of elasticity and viscoelasticity, of hydrogels for biological applications. To do so, poly(ethylene glycol) diacrylate (PEGDA) hydrogels were fabricated with different PEG chain lengths and polymer concentrations, and their mechanical properties were characterized via oscillatory rheology, uniaxial compression, and AFM. By cross-evaluating the results of different techniques, insight into the correlations and discrepancies between them is provided. Solid-state nuclear magnetic resonance (ssNMR) was used to assess the molecular mobility. These hydrogels were covalently grafted with a mixture of an adhesion peptide (RGD) and an osteogenic peptide (BMP-2 mimetic peptide). The covalent grafting, peptide density, and homogeneous distribution were verified through X-ray photoelectron spectroscopy and fluorescence microscopy. Finally, hMSCs were cultured on these materials and assessed for osteogenic differentiation, demonstrating that the investigated PEGDA biofunctionalized hydrogels can support osteogenic differentiation in cells cultured on a basal medium.

## Materials and Methods

### Materials

Poly(ethylene glycol) diacrylate (PEGDA, *M*_w_ 4000 Da), 2-hydroxy-4′-(2-hydroxyethoxy)-2-methylpropiophenone (Irgacure 2959), 4-(2-hydroxyethyl)-1-piperazineethanesulfonic acid (HEPES), paraformaldehyde, Triton X-100, Tween 20, and bovine serum albumin (BSA) were obtained from Sigma-Aldrich (France). Poly(ethylene glycol) diacrylate (PEGDA, *M*_w_ 400 Da) was obtained from PolyScience (Pennsylvania, USA). Phosphate buffered saline (10×) (PBS), trypsin/EDTA (ethylenediaminetetraacetic acid), penicillin/streptomycin, fetal bovine serum (FBS), Dulbecco’s modified Eagle medium (DMEM), DAPI (4′,6-diamidino-2-phenylindole, dihydrochloride), Alexa Fluor 488 phalloidin, goat anti-mouse lgG (H + L) highly cross-adsorbed secondary antibody Alexa Fluor 647, and goat anti-rabbit lgG (H + L) highly cross-adsorbed secondary antibody Alexa Fluor 647 were purchased from Thermo Fisher Scientific (USA). Rabbit anti-RUNX2 primary antibody was acquired from Cell Signaling (USA). Mouse monoclonal antiosteopontin was obtained from Santa Cruz Biotechnology (USA). Mouse monoclonal antipodoplanin/E11 was obtained from Abnova (U.K.). Bone marrow-derived hMSCs and MSC osteogenic differentiation medium were obtained from Promocell (Heidelberg, Germany). CKIPKASSVPTELSAISMLYLK(FITC), KRKIPKASSVPTELSAISMLYLC, and CG-K(PEG3-TAMRA)-GGRGDS peptides were synthesized by Genecust (Boynes, France). Silicon isolators were obtained from Grace Bio-Laboratories (Oregon, USA).

### Hydrogel Fabrication

The mechanical properties of the PEGDA hydrogels were controlled by mixing polymer molecules with two different chain lengths at different concentrations in aqueous media. Accordingly, the appropriate amount of poly(ethylene glycol) diacrylate (PEGDA) (*M*_w_ = 4 kDa and 400 Da) was dissolved at different ratios in PBS to form solutions with a total polymer content of 5, 10, 15, 20 or 30% w/v. The nomenclature indicates first the weight ratio of long- to short-chain polymer content, followed by the total polymer concentration (e.g., a 20/80 20% material is formed with 20% w/v total polymer concentration, of which 20% is PEGDA 4 kDa and 80% is PEGDA 400 Da). After dilution, 0.7 wt % of the photoinitiator Irgacure 2959 was added from a stock solution of 0.1 g/mL in ethanol. The polymer solution was quickly vortexed and pipetted into silicone wells (9 mm diameter, 1.7 mm thickness) sandwiched between two glass coverslips coated with a hydrophobic fluorinated ethylene propylene sheet. Gel formation was induced with a UV lamp (Uvitec, U.K.) for 15 min (365 nm, 64 W) (Figure S1A). The resulting hydrogels were then thoroughly rinsed in PBS and left to swell in PBS at 4 °C for at least 24 h before any testing.

### Swelling

Hydrogel discs were weighed at equilibrium swelling in PBS. The samples were then frozen at −80 °C overnight, lyophilized for 24 h and weighed again. Fluid absorption capacity was calculated as the ratio of the difference between the swollen and dry mass over the dry mass. Measurements were performed in triplicate.

### Compression

Compression tests were performed in a TA Discovery Hybrid Rheometer 10 equipped with an axial force transducer. Hydrogel discs of 8 mm were placed on the stage and compressed at a rate of 1 mm·min^–1^ with a flat 8 mm plate until 10% strain was reached. A preload of 2 mN was applied before the tests. Stress–strain curves were plotted, and the slope of the linear fit in the 0–10% strain region is used as the measure for elastic modulus. Three samples of each condition were analyzed.

Relaxation tests were performed in a PBS bath using a Mach-1 V500CS (Biomomentum, Canada) mechanical tester equipped with a 1.5 N single-axis load cell. Hydrogel discs of 10 mm diameter were placed in the middle of the stage and compressed with a flat 10 mm plate until a preload of 2 mN was reached. Then, a compression step of 5% strain was applied at a rate of 1 mm·min^–1^. The deformation of 5% strain was held constant while the load was recorded as a function of time for 5 min. The relaxation degree (%) was calculated as the proportion of the initial stress that has been lost at the end of the relaxation period.

### Shear Rheology

Rheological measurements were performed on an Anton Paar MCR 302 rheometer equipped with a sand-blasted parallel plate geometry (25 mm diameter). Hydrogel discs were prepared with a diameter of 25 mm and a thickness of approximately 1 mm. Samples were kept in place between the plates by inducing a normal force of 0.5 N at the beginning of each test. The temperature was kept at 25 °C. First, to determine the linear viscoelastic region, a strain sweep was performed on a specimen of each hydrogel composition at a frequency of 1 Hz over the 0.1–10% strain range. Storage (*G*′) and loss (*G*″) moduli were measured from strain sweeps at 0.5% strain with a frequency of 1 Hz. Stress-relaxation experiments were performed by inducing a shear strain of 1% and recording the resulting stress until half of the initial value was reached. Measurements were repeated on a minimum of 3 samples.

### Atomic Force Microscopy

AFM characterization was done in an MFP-3D-BIO atomic force microscope (Asylum Research, Oxford Instruments, USA), using silicon nitride cantilevers with a colloidal polystyrene tip of 10 μm diameter and a nominal spring constant of 0.6 N·m^–1^ (Novascan, USA). Prior to testing, fully hydrated hydrogels were glued to a glass slide and the cantilevers were calibrated by the thermal method. All tests were performed in fluid immersion conditions (PBS) at room temperature.

For elasticity measurements, force–indentation curves were obtained with an approach and retract speed of 2 μm·s^–1^ and a force set point of 2 nN. Measurements were performed on 3 samples per condition, with a minimum of 100 curves analyzed per sample, acquired in 3–4 randomly selected areas of each material. Young’s moduli values were calculated by fitting the extend curve to the Hertz contact model for a spherical indenter with the Asylum Research analysis software, assuming a sample Poisson ratio of 0.5.

In relaxation experiments, similar indentations with an approach and retract speed of 2 μm·s^–1^ and a force set point of 2 nN were done, maintaining a constant *z* height during 40 s between the approach and retract portions. Three samples per condition were studied, with at least 10 measurements per sample from different areas of the surface. Relaxation percent was evaluated with a custom MATLAB script, by calculating the proportion of the force after 40 s of relaxation to the maximum force at the end of the approach portion.

Microrheology tests consisted of a relaxation segment at a constant *z* height of 5 s after the indentation step, followed by an oscillation segment, in which the probe was directed to move in a sinusoidal pattern with an amplitude of 20 nm and frequencies ranging from 0.5 to 40 Hz for 30 s.^26,27^ These measurements were performed on two to three independent samples, with at least 10 curves analyzed per sample. Analyses were performed with a custom MATLAB script. Briefly, the *z* position and force signals corresponding to the oscillation segments were smoothed by using a bandpass Butterworth filter with appropriate cutoff frequencies for each oscillation frequency. Then, each oscillation cycle was isolated and the phase lag (Δ) between the force and displacement spectra was calculated with the equation proposed by Lai and Hu:^26^
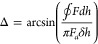
where *F*(*d*) is the recorded force as a function of the tip displacement, δ*h* is the displacement amplitude, and *F*_a_ is the amplitude of the force response.

### Solid-State NMR

Magic-angle spinning (MAS) solid-state NMR experiments were performed on a 600 MHz ^1^H Larmor frequency spectrometer (Bruker Biospin) using a 4 mm triple resonance HCN MAS probe head. Solid-state NMR rotors were filled with approximately 5 mg of sample, and a MAS frequency of 11 kHz was used. The sample temperature was adjusted to 7 °C for the low-temperature experiment, according to the DSS signal used as an internal reference.^28^ A ramped cross-polarization (CP) of 1 ms was used for the ^1^H–^13^C polarization transfer in the CP experiments. An acquisition time of 20 ms was used for ^1^H–^13^C CP, INEPT, and ^13^C DP experiments. High-power proton decoupling was applied using a SPINAL-64 decoupling sequence during detection.^29,30^ All data were processed and analyzed using Topspin 4.1.3 (Bruker Biospin).

### Peptide Functionalization and Fluorescence Microscopy

The hydrogels were functionalized with either a single peptide or a 1:1 solution of two peptides, an adhesion peptide (RGD) and an osteogenic promoter (BMP-2 mimetic peptide), using the heterobifunctional cross-linker sulfoSANPAH, as described in previous publications.^25^ Briefly, the hydrogels were soaked in a 1 mM sulfoSANPAH solution in HEPES buffer (pH 8.5, 50 mM) and exposed to UV light (365 nm, 64 W) for 15 min. The excess solution was removed, and the process was repeated after turning the hydrogels over to the other side. Then they were rinsed twice with HEPES before being immersed in a peptide solution (total peptide concentration of 0.5 mM in HEPES) and incubated overnight under agitation. The gels were then rinsed in HEPES for 3 days under agitation to remove the adsorbed peptides.

The peptide-grafted materials were characterized using fluorescence microscopy (ZEISS LSM800 confocal laser scanning microscope using Zen Blue software (Zeiss, Germany)). The hydrogels were functionalized with a 1:1 molar solution of a BMP-2 mimetic peptide linked to an FITC fluorochrome at the C-terminal of the lysine amino acid (BMP-2-FITC) and an RGD peptide containing a TAMRA fluorochrome (RGD-TAMRA). The RGD peptide sequence contains a three-unit ethylene glycol spacer linked to the side chain of a lysine amino acid to which the TAMRA molecule is bound (CG-K(PEG3-TAMRA)-GGRGDS). Images of the functionalized materials were acquired at 5× magnification with a zoom of 0.5 (Objective 5×/0.16, 2048 × 2048, 16 bits per pixel). BMP-2-FITC and RGD-TAMRA materials were imaged with lasers of 488 and 561 nm, respectively, with a pinhole aperture of 1 airy unit. Eight images from two independent materials of each condition were acquired. Similar images were acquired of nonfunctionalized materials to have a measure of the fluorescence background intensity. A series of 1 μL drops of the two peptide solutions at different concentrations (15–75 μM) were deposited on polyethylene terephthalate films and imaged with the same parameters as the hydrogels to obtain a calibration curve for each peptide, which was then used to measure the amount of peptide on the gel surface. The fluorescence intensity of the microscopy images was quantified using the ImageJ freeware.

### X-ray Photoelectron Spectroscopy

After each step of peptide grafting, surface chemical compositions were determined by XPS. For these characterizations, the functionalized gels contained a single peptide, either RGD or BMP-2 fluorescent peptides. Only hydrogels of composition 100/0 10% were analyzed by this technique. Samples were removed from water and placed immediately on the XPS sample holder, where they were dehydrated under a vacuum.

Hydrogels were analyzed using a Thermo Fisher Scientific K-ALPHA spectrometer with a monochromatized Al–Kα source (*h*ν = 1486.6 eV) and a 400 μm X-ray spot size. Four measurements per sample were carried out to ascertain the reproducibility of the surface chemistry. The survey spectra (0–1100 eV) were recorded using a constant pass energy of 200 eV, while high-resolution spectra were recorded with a continuous pass energy of 40 eV. Charge neutralization was applied during the analysis. High-resolution spectra (i.e., C 1s, O 1s, N 1s, and S 2p) shifted versus the main C 1s component at around 286.3 eV were quantified using Avantage software provided by Thermo Fisher Scientific. C 1s, N 1s, and S 2p HR spectra were curve-fitted to ascertain the chemical environments of these atoms.

### Cell Culture

Materials for the cell culture were functionalized with a 1:1 solution of RGD-TAMRA and BMP-2. For cell culture experiments, the materials were sterilized by soaking for 5 h in ethanol 70% w/v, followed by rinsing 3 times and soaking overnight in sterile PBS. The materials were immersed in serum-free medium for 2 h, before placing them in wells of a sterile 48-well plate. Bone marrow-derived human mesenchymal stem cells (hMSCs) on passage 5 were seeded in DMEM at a density of 2500 cells per cm^2^ on the hydrogels and glass slide controls and kept in an incubator at 37 °C and 5% CO2. The medium was supplemented with 1% antibiotic solution. For the 24 h adhesion experiment, cells were kept in serum-free media until fixation. For the 1 and 2-week experiments, 10% FBS was added after 4 h of incubation. In the case of experiments with an osteogenic induction medium, the differentiation medium was added after 24 h. Cells were kept in culture for 1 or 2 weeks, with the medium changed every third day. Cells for controls were seeded at the same density on glass coverslips and kept in culture with either DMEM + 10% FBS (referred to as “C-DMEM”) or osteogenic differentiation medium (“C-OM”).

### Immunofluorescence Staining

For the evaluation of the cell spreading area after 24 h of culture, cells were fixed using 4% paraformaldehyde solution at 4 °C for 15 min, permeabilized with 0.5% TritonX-100 for 5 min, and saturated with 3% bovine serum albumin for 1 h at 37 °C in a humid atmosphere. Afterward, the cytoskeleton was stained by incubating with Alexa Fluor 488 phalloidin (1:40 dilution) for 1 h at 37 °C, and the nuclei were marked with DAPI (1:1000 dilution). Stained cells were examined using a Leica DM5500B epifluorescence microscope (Leica Biosystems) equipped with a CoolSnap HQ camera and controlled by Metamorph 7.6 software. Three samples per condition were imaged, with at least 40 cells per condition being analyzed.

In the differentiation experiments, cells were fixed after 1 or 2 weeks of culture. They were permeabilized with 0.5% TritonX-100 for 5 min and saturated with 3% bovine serum albumin for 1 h at 37 °C in a humid atmosphere. Afterward, the samples were incubated with the primary antibody diluted in 1% BSA/PBS for 2 h at 37 °C in humid atmosphere (anti-Runx2 at a dilution of 0.25 μg·mL^–1^, anti-OPN at a dilution of 1 μg·mL^–1^, and anti-E11 at a dilution of 2 μg·mL^–1^). Each material was then incubated with the corresponding secondary antibody, goat antimouse lgG (H + L) highly cross-adsorbed secondary antibody Alexa Fluor 647 or goat antirabbit lgG (H + L) highly cross-adsorbed secondary antibody Alexa Fluor 647, diluted at 5 μg·mL^–1^ in 1% BSA/PBS for 1 h at 37 °C in a humid atmosphere. The cell cytoskeleton was marked by incubating with Alexa Fluor 488 phalloidin for 1 h at 37 °C. Finally, cell nuclei were stained using DAPI at a 1:1000 dilution. All samples were washed with PBS containing 0.05% Tween 20 between the different incubation steps.

The stained cells were examined using a Leica DM5500B epifluorescence microscope (Leica Biosystems) equipped with a CoolSnap HQ camera and controlled by Metamorph 7.6 software. Images to evaluate cell distribution on the surfaces and cell morphology were acquired at 10× and 40× magnification. Cell area, Aspect ratio (AR) and nuclear area were evaluated by delineating the contour of the cells or the nuclei in the fluorescence images. AR corresponds to the ratio of the major to minor axes of the best-fit ellipse of a cell calculated using ImageJ software. Morphological measurements were performed on two independent experiments with two samples per condition for each experiment. Between 40 and 60 cells are evaluated for cell area and AR, and more than 100 cells are evaluated for nuclear area. The protein marker expressions were assessed in 40× magnification images using ImageJ. The expression of Runx2 and osteopontin was evaluated by quantifying the total fluorescence intensity per nuclei, on a total of 60–100 cells with two samples per condition. The expression of E11 was considered on the whole cell, on a total of 40–60 cells with two samples per condition. A background signal equivalent to each measured area was subtracted from all measurements.

### Statistical Analyses

Data are expressed as mean values ± standard deviation. Statistical analyses for the mechanical data were performed using multiple *t* tests, with Welch’s correction in case of unequal variance, with GraphPad Prism 8.0.1 for Windows. For AFM data, the absence of normality is verified with GraphPad Prism in all cases. As a result of this, nonparametric Kruskal–Wallis tests are performed for AFM mechanical data. For the data of functionalization, cell morphology, and protein expression, significance was assessed by one-way ANOVA with Tukey’s correction for multiple comparisons. Significant differences were determined for *P* values ≤0.05 (with *, **, ***, and **** representing *P* < 0.05, *P* < 0.01, *P* < 0.001, and *P* < 0.0001, respectively).

## Results and Discussion

### Characterization of the Elastic Behavior of PEGDA Hydrogels

This study involves producing hydrogels by end-cross-linking solutions of PEGDA through photoinitiated free radical polymerization. Physical chain entanglements and hydrogen bonding also contribute to the network.^31^ PEGDA hydrogels were synthesized by varying the total polymer concentration and molecular weight of the initial PEGDA chains (400 Da and 4 kDa) to obtain scaffolds with different compositions.

**Figure 1:**
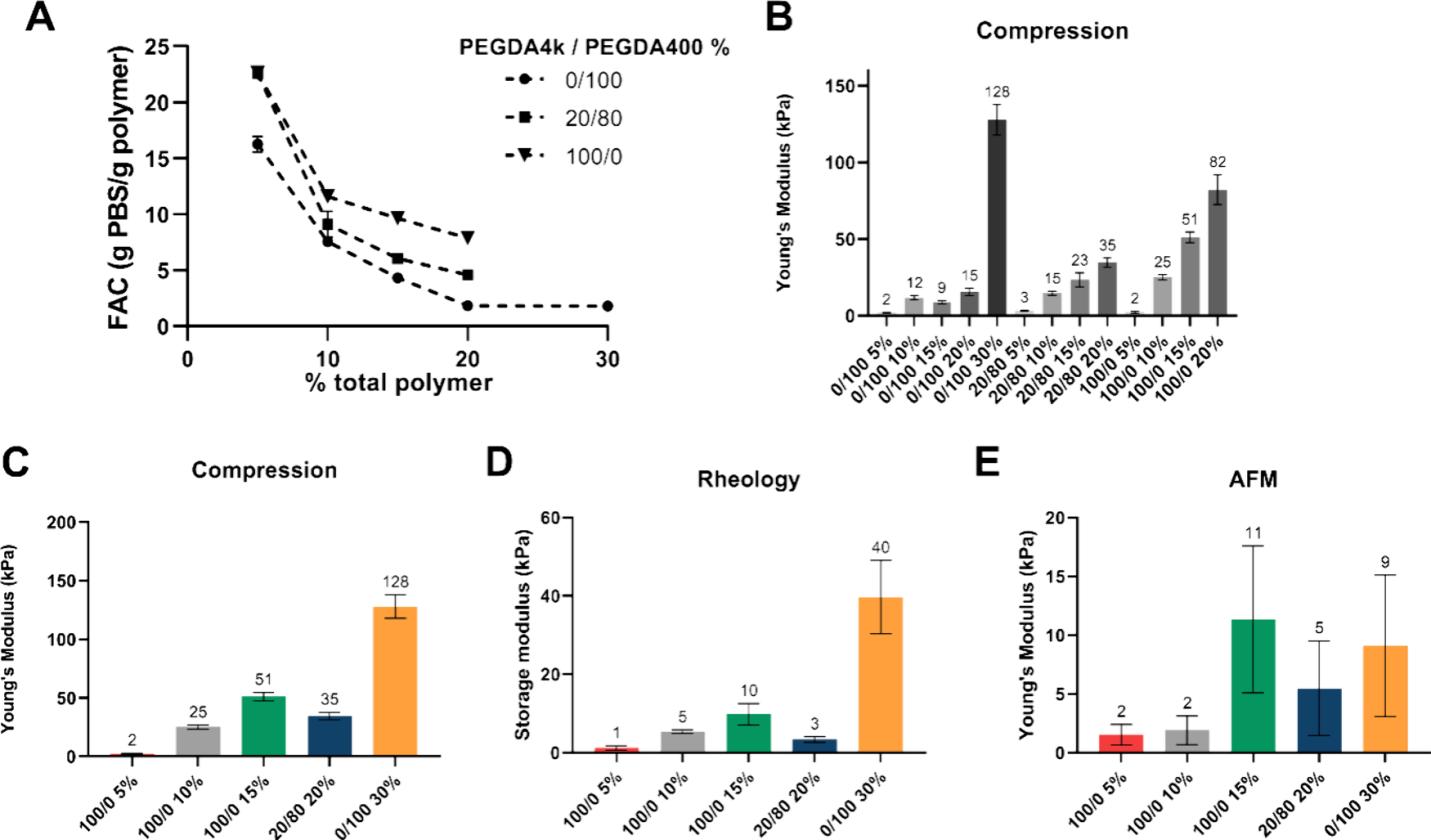
Mechanical evaluation of the elasticity of hydrogels. (A) Fluid absorption capacity (grams of PBS per gram of polymer) as a function of total polymer concentration for materials fabricated with different ratios of 4 kDa and 400 Da PEGDA. (B) Young’s modulus measured from macroscopic compression tests of all hydrogel compositions. (C–E) Evaluation of the elasticity of 5 selected hydrogels, with (C) macroscopic compression, (D) rheology, and (E) AFM. The statistical analysis data are presented in the Supporting Information for clarity (Tables S1 and S2).

The network density of hydrogels with different compositions was studied by looking at their swelling properties (Figure 1A). These hydrogels can absorb large amounts of water, from 2 to more than 20 times their own dry weight. Fluid absorption capacity (FAC) is reduced as the total polymer concentration increases, which leads to a denser network that can absorb less liquid. As for the ratios of long to short chain polymer, it is noticeable that hydrogels composed of short chains (0/100) have less FAC than those composed of long chains (100/0), again due to the differences in network density since, for a given mass concentration, the higher molecular mass results in a decreased number of end-of-chain acrylate groups that are available for cross-linking.^32^ In turn, those composed of a mixture of long and short chains (20/80) have an intermediate FAC. As shown in Figure S2, after 24 h, all hydrogels are fully swollen. These results are in agreement with those observed for similar hydrogels of poly(ethylene glycol) dimethacrylate^33^ or gelatin-PEGDA.^34^

The elastic modulus of all the different hydrogel compositions was characterized by uniaxial unconfined compression (Figure 1B). For a given ratio of chain lengths, increasing the polymer concentration results in hydrogels with increasing Young’s Modulus, from 3 to 35 kPa for the 20/80 samples, and 2–82 kPa for the samples composed only of long PEGDA chains (100/0). Interestingly, in the case of the samples composed of short chains (0/100), Young’s modulus does not significantly increase when the concentration is raised from 10 to 15 and 20% (12, 9, and 15 kPa respectively), but it increases dramatically up to 128 kPa when the polymer concentration grows to 30%. This is likely because the short PEGDA chains at relatively low concentrations dispersed in a solvent are not able to form a fully reticulated network and rather the elongation of the chain is prioritized, with multiarm cross-links occurring only at scattered locations throughout the scaffold.

The elastic behavior of five selected samples was further characterized using oscillatory shear rheology and AFM with a colloidal tip (nominal diameter 10 μm) (Figure 1C–E). Figure 1C shows the Young’s modulus measured under macroscopic compression, discussed previously. In the samples composed solely of long PEGDA chains (100/0 hydrogels), the stiffness increases with increasing polymer concentration, with moduli ranging from 2 up to 51 kPa. The stiffest composition tested (0/100 30%), composed only of short PEGDA chains at 30% w/v, has an average modulus of 128 kPa. The sample 20/80 20%, which contains a mixture of short and long chains, has an average Young’s modulus of 35 kPa.

The rheological characterization allows to calculate the storage modulus (*G*′) (Figure 1D), which is a measure of the energy that is conserved during the oscillations and is indicative of the elastic behavior of the sample. For the hydrogels composed of long PEGDA (100/0 materials), the storage modulus increases as the polymer concentration increases, ranging from 1.2 ± 0.6 up to 10 ± 3 kPa. The materials fabricated with a high concentration of short PEGDA chains (0/100 30%) are the stiffest, with a modulus of 40 ± 9 kPa for the material 0/100 30. Interestingly, the hydrogel with 20% long-chain PEGDA and 80% short-chain PEGDA (but with a total polymer concentration of 20%) has a modulus of 3.4 kPa. This observed shear modulus deviates from our initial expectations; however, the reproducibility was verified by testing 5 materials from different batches. Despite the presence of short chains and a polymer concentration of 20%, this hydrogel displays a relatively low stiffness, which is similarly observed in compression and AFM. Mazzoccoli et al.^35^ showed that, at a concentration of 20%, blending PEGDA 400 and 3400 Da had a small effect on the measured compressive modulus. The microstructure of hydrogels in general and PEGDA hydrogels in particular tends to be heterogeneous. It has been suggested that the network of PEGDA hydrogels consists of highly cross-linked points that are connected by longer and less dense polymer chains.^31,36^ In addition, the distribution of the network of PEGDA hydrogels composed of chains with large differences in molecular weight may present distinct structures^37^ that result in such values of elastic modulus lower than expected.

It can be observed that a general trend in the values is maintained between the macroscale measurements of *G*′ and Young’s modulus for each of the samples. A discrepancy is observed for the hydrogels 20/80 20%, which appear to be stiffer than the samples with composition 100/0 10% in compression but softer than those same ones under rheological testing. Pizzolitto et al.^38^ also noted discrepancies in the *G*′ and Young’s modulus of lactose-modified chitosan hydrogels, which are attributed to changes in the proportion of elastically active segments. In addition, the calculated *E*/*G*′ ratio is not constant across the 5 samples, suggesting nonaffine network deformation.^39^ These data demonstrate that while these different testing modalities for the elastic modulus provide a relative indication of the stiffness of the samples, each technique subjects the material to different deformation regimes and the behavior of the polymer chains is not equivalent. A similar phenomenon was noted by García-Abrego et al.,^39^ who tested PEG and fibrin hydrogels and reported deviations in this ratio, depending on the intrinsic hydrogel porosities and mesh sizes.

AFM indentation, with either pyramidal or colloidal tips, is a well-established method to evaluate the mechanical properties of hydrogels for tissue engineering. Figure 1E summarizes the calculated Young’s modulus for each hydrogel composition, measured by AFM with a colloidal tip. Similarly to the bulk measurements of elastic modulus performed in macroscopic compression, the modulus of 100/0 (long PEGDA chains) hydrogels increases with increased polymer concentration, from 1.5 up to 11 kPa for samples at 5 and 15% respectively. The 20/80 20% had an intermediate value of 5 kPa. These values measured using AFM are, in all cases, lower than the ones calculated by compression. It must be noted that other authors have shown that changes in the AFM measurement parameters employed can result in drastically different results. For instance, García-Abrego et al.^39^ characterized PEG and fibrin hydrogels with AFM indentation using pyramidal and colloidal tips and consistently found higher values of elastic modulus when using the pyramidal indenters, from 4 to 30 times higher, depending on hydrogel composition.

A limited number of investigations have been conducted on the comparison of hydrogel mechanical properties measured by different techniques. A review study by Oyen^40^ aimed to compile results from different publications on mechanical testing of agar and polyacrylamide hydrogels. The comparison shows very large and not systematic discrepancies, with up to 2 orders of magnitude differences for both hydrogel types. This questions whether results from studies that have used different testing modalities can be set side by side. However, in this case, comparison between studies of different authors brings into play not only variations due to the testing method but also potential disparities in the fabrication of the materials or reproducibility issues. Richbourg et al. characterized poly(vinyl alcohol) hydrogels and found some discrepancies, especially for soft hydrogels with modulus under 20 kPa.^41^ Despite the global equivalency found among all techniques, the highest variability is attributed to the nanoindentation method due to the large number of parameters and assumptions involved. Indeed, a study showed that the indenter size, indentation speed, and thickness of the sample influence Young’s Modulus values obtained for soft hydrogels.^42^ The results presented in this work demonstrate that while the general tendencies are maintained across the different techniques, the numerical values are not always maintained, and thus one has to be careful when comparing results obtained with different measurement strategies.

### Characterization of Viscoelastic Properties of PEGDA Hydrogels

Viscoelasticity, the time-dependent mechanical behavior of materials, can be measured with different techniques and through different types of testing modalities. One of the most common approaches to studying viscoelasticity is via stress-relaxation tests, either reporting the fraction of stress dissipated after a certain period of relaxation^14,25^ or, more commonly, the relaxation half-time.^43,44^ In other cases, materials are subject to dynamic testing and the loss modulus or loss tangent is used as an indication of viscoelastic behavior.^13,38^ In addition, these tests can be performed at different scales and deformation modalities, commonly through rheology, compression testing, or AFM-based indentation. Despite this variety of characterization techniques for the viscoelasticity of hydrogels, no studies that aim to compare and contrast the differences among them have been found.

**Figure 2:**
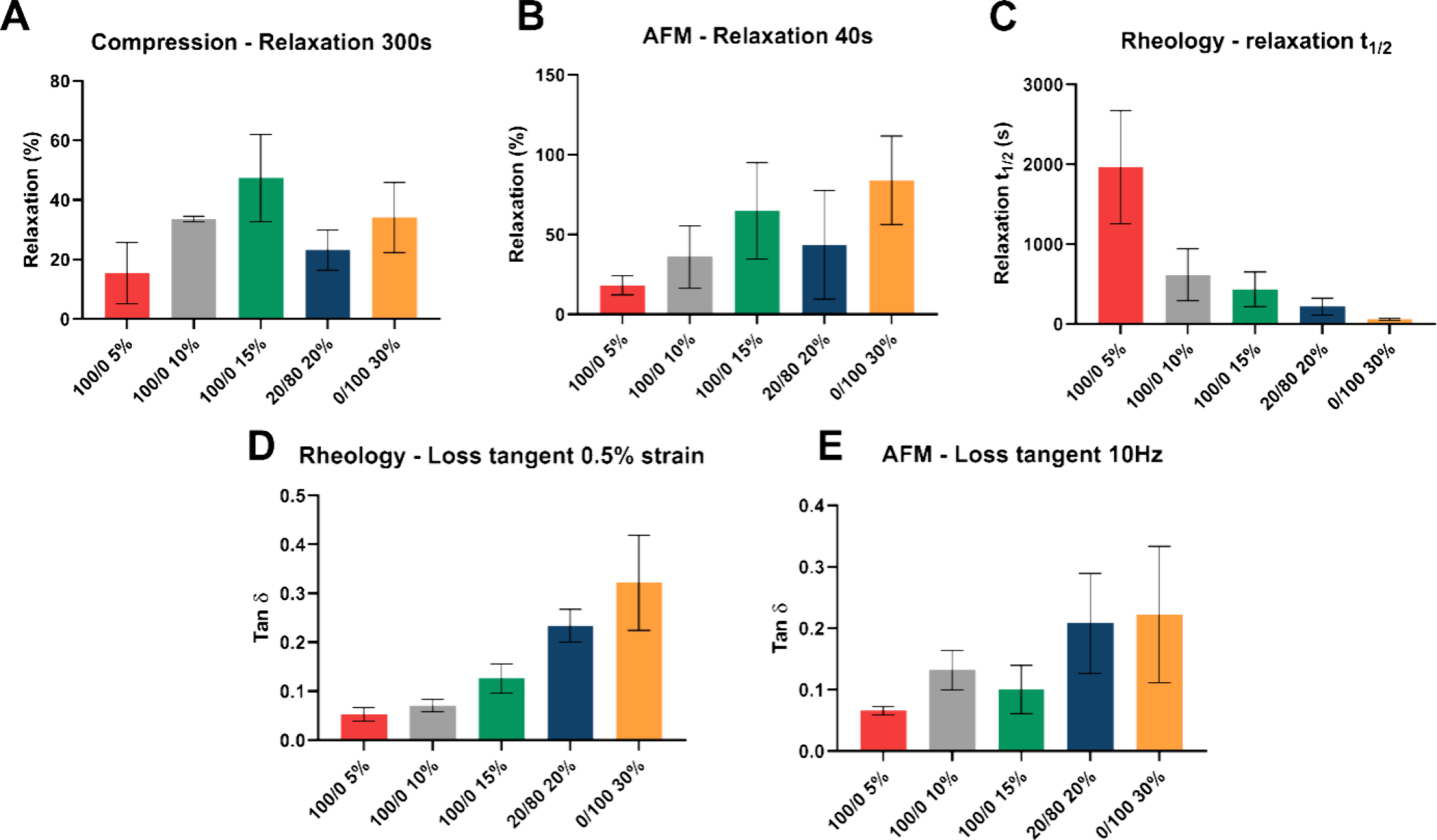
Viscoelastic characterization of different PEGDA hydrogels. (A) Percent stress-relaxation after 300 s under macroscopic compression. (B) Percent stress-relaxation after 40 s under AFM indentation. (C) Time scale of stress-relaxation, τ_1/2_, defined as the time that it takes for the maximum stress to be reduced to half of its original value, under shear rheology. (D) Loss tangent calculated from shear rheology. (E) Loss tangent calculated from the AFM microrheology. The statistical analysis data are presented in the Supporting Information for clarity (Table S3).

In this work, the viscoelasticity of five different PEGDA hydrogels has been evaluated with stress-relaxation tests in compression, AFM microindentation, and rheology, and with dynamic testing via rheology and AFM (Figure 2). It is clear with all techniques that the viscoelasticity of the hydrogel samples changes from one composition to another, and, despite some discrepancies, common tendencies can be observed regardless of the testing modality.

Macroscopic compression stress-relaxation tests reveal distinct viscoelasticity across different PEGDA hydrogels (Figure 2A). The relaxation behavior is given by the percent of the initial stress that is lost during a relaxation period of 300 s. In the case of 100/0 compositions, the hydrogels at 5% concentration exhibited the lowest relaxation with an average of 15%, while the more concentrated hydrogels at 10% and 15% displayed relaxation percents of 33 and 47, respectively. The hydrogel with short chains (0/100 30%) has an average relaxation of 34%, while the measured relaxation of the sample 20/80 20% is 23%. A similar measurement of relaxation was performed at the surface level with AFM (Figure 2B). The absolute values are challenging to compare, since the length scales and time scales, as well as the forces applied in each case, are very different. However, it is notable that the tendencies are comparable, with the least relaxing composition being 100/0 5% and the highest relaxation values found for samples 100/0 15% and 0/100 30%. Only a few other publications^45−48^ have used AFM to characterize surface stress-relaxation of hydrogels, and, to the best of our knowledge, no reports comparing the macro- and microscale relaxation behavior of biomedical hydrogels have been found. Given that cells are able to probe the mechanical properties of ECM within a radius of around 5 μm around them^8,49^ and they exert forces on the order of nanonewtons,^50^ it is important to ascertain whether the viscoelastic properties measured at the macroscale can be extrapolated to those presented to cells at the microscale. Our results show that for the PEGDA blends of interest in this study, the relaxation behavior under macroscopic compression is comparable to that observed at the surface level in AFM.

Another commonly used method to study viscoelastic materials is rheology. Similar stress-relaxation tests, under shear deformation in this case, were conducted for the same PEGDA blends. The parameter τ_1/2_, denoting the duration required for the stress within a hydrogel to decrease to half of its initial magnitude, exhibited varying values across gel types (Figure 2C). τ_1/2_ was found to decrease with increasing total polymer concentration, regardless of the ratios of long to short chains, although the differences between materials 100/0 10, 100/0 15, and 20/80 20% were not found to be statistically significant (at the level of *p* ≤ 0.05, Table S3). The results for the long-chain PEGDA samples follow the tendency observed in compression and AFM, with relaxation increasing with the polymer concentration. In contrast, samples 20/80 20 and 0/100 30% appear to have a higher relaxation under rheology than with the two other techniques.

Finally, dynamic testing was performed on different hydrogel compositions at the macroscale with rheology and at the microscale with AFM. Along with stress-relaxation, dynamic testing is another commonly used method to study viscoelasticity, generally reporting loss modulus (*G*″) or loss tangent (tan(δ)), with higher values of these parameters indicating a higher proportion of viscous behavior. Loss tangent measured in rheology (Figure 2D) is in agreement with the static rheological testing previously discussed. The application of dynamic testing with AFM, also sometimes termed microrheology, is still not widely used in the biomaterials field. Here, the loss tangent of different materials is calculated from the phase lag between the indentation depth and the force response originated in dynamic indentation tests, as proposed by Lai and Hu.^26^Figure 2E shows the calculated loss tangent at a testing frequency of 10 Hz. The results in the full range of frequencies tested (0.5–40 Hz) are shown in Figure S4. The loss tangent values obtained range from 0.07 to 0.22, which are similar to those obtained by other authors for similar hydrogels.^27,51^ This method of AFM microrheology enables us to obtain the microscale equivalent of the classic dynamic rheological testing. In the case of this PEGDA hydrogel, some differences can be observed. Notably, with AFM, 100/0 15% hydrogels have a lower tan(δ) compared to 100/0 10% ones, and there is no significant difference between the tan(δ) of 20/80 20 and 0/100 30% samples. These differences could be attributed to different behaviors at the surface versus those at the bulk of the material. However, the dispersity of measurements in AFM is large and does not allow us to conclude beyond any doubt.

Overall, good agreement was found among all of the viscoelasticity characterization techniques described here. Specifically, stress-relaxation under compression and AFM yielded highly similar results, a significant finding, given the scarcity of literature comparing the surface and bulk viscoelasticity in biomedical hydrogels. Furthermore, rheological measurements, whether assessing stress relaxation or loss tangent, exhibited consistent outcomes, affirming the reliability of both static and dynamic rheological methods. In addition, we performed AFM microrheology, which is a novel technique that enables dynamic testing at the surface level.

### Solid-State NMR

**Figure 3:**
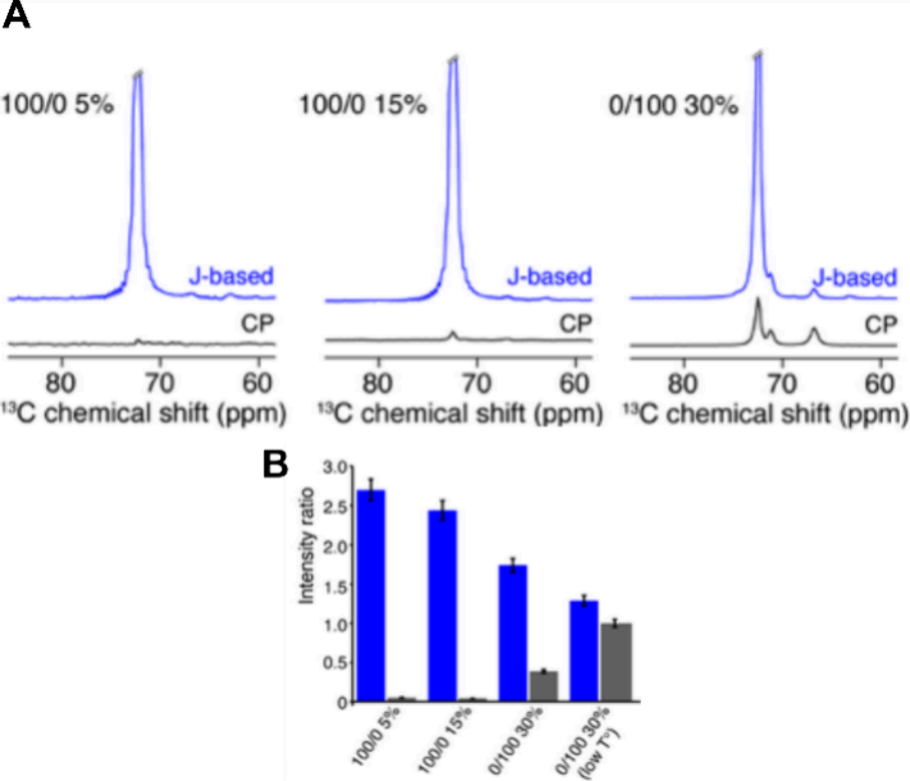
Solid-state NMR characterization of hydrogel mobility. (A) INEPT (in blue) and CP (in black) ^13^C-detected spectra obtained at 600 MHz at a magic-angle spinning frequency of 11 kHz. (B) Intensity ratio between INEPT and direct polarization spectra (in blue) and between CP and DP (in black) recorded at room temperature. Low temperature is 280 K.

To gain insights into the molecular origin of the mechanical properties, three hydrogels with different mechanical behavior were analyzed by magic-angle spinning (MAS) solid-state NMR. Two types of polarization transfer were employed at room temperature to distinguish different regimes of molecular mobility. 100/0 5, 100/0 15, and 0/100 30% hydrogels presented a very intense signal using J-based INEPT (Figure 3A in blue), this type of polarization transfer probing highly mobile chains. Much weaker signals were observed using cross-polarization (CP) transfer (Figure 3A in black), which reveals rigid and immobilized chains. To assess more quantitatively the sample mobility observed between the three gels, INEPT and CP ^13^C spectra were compared to direct polarization (DP) ^13^C spectra by measuring the intensity ratio (Figure 3B). A gradual decrease of the INEPT/DP ratio was observed when the polymer concentration increased, indicating that the polymer chain mobility is restricted at higher concentrations. For 100/0 5 and 100/0 15% gels, a CP/DP ratio close to 0 was measured, suggesting the absence of an immobilized chain. A substantial increase is observed for the 0/100 30% hydrogel (CP/DP ratio of approximately 0.4), resulting from a rigidification of polymer chains. This hydrogel was also tested at a lower temperature (280 K), which led to observe a global loss of mobility (higher CP/DP ratio and lower INEPT/DP ratio), presumably resulting from lower thermal fluctuations occurring at 280 K compared to room temperature.

These findings suggest that there is a correlation between the chain mobility and bulk elasticity of the PEGDA hydrogels with different compositions. As observed in Figure 3B, the chain mobility, encoded in the INEPT/DP ratio (blue bars) was the highest for the gel 100/0 5% gel and the lowest for the 0/100 30% gel, with an intermediate value for 100/0 15%. This is inversely proportional to the storage modulus measured in rheology (Figure 1D). Remarkably, a slight change in the polymer concentration (from 15 to 30%) leads to a drastic increase in the number of immobilized chains as measured by CP experiments. This is indicative of a very restrained mobility despite only 30% polymer content in the hydrogel, also reflected in the low intensity of the INEPT spectrum. This suggests that potential fine-tuning of internal chain dynamics can be obtained from a simple change in polymer concentration. The importance of studying the mechanical properties of cell culture scaffolds at different scales has been recently recognized;^41^ however, very few publications address these properties at the molecular scale. A recent publication shows that ssNMR analysis of chain mobility can be correlated with rheological measurements of peptide hydrogels.^52^ The observed increasing intensity in the CP spectrum correlated to an increasing polymer content in the hydrogel is probably due to molecular packing, restraining the molecular motion. The integrative use of ssNMR together with rheological measurements might offer a promising route to bridge the gap between macroscopic mechanical and microscopic molecular properties of soft biological materials and ultimately to improve the design of biomaterial scaffolds for cell culture.

### Evaluation of Hydrogel Functionalization with Bioactive Peptides

PEGDA hydrogels are highly resistant to protein adsorption^24^ and necessitate the incorporation of adhesive ligands to enable cellular attachment and further influence cell fate. To favor adhesion, spreading, and osteogenic differentiation of cells, the hydrogels were covalently bound with two peptides, an RGD peptide and a peptide derived from the sequence of the BMP-2 protein.^9,19−21^ In this work, the heterobifunctional cross-linker sulfoSANPAH was used to covalently bind the peptides to the hydrogel, as previously described in the literature.^24,25^ On one side of the cross-linker molecule, the nitrophenylazide is activated under UV light and inserted into C–H sites in the polymer through click chemistry. The next reaction step involves the reaction of the succinimidyl ester on the other side of the cross-linker molecule with the primary amines of the peptide.

**Figure 4:**
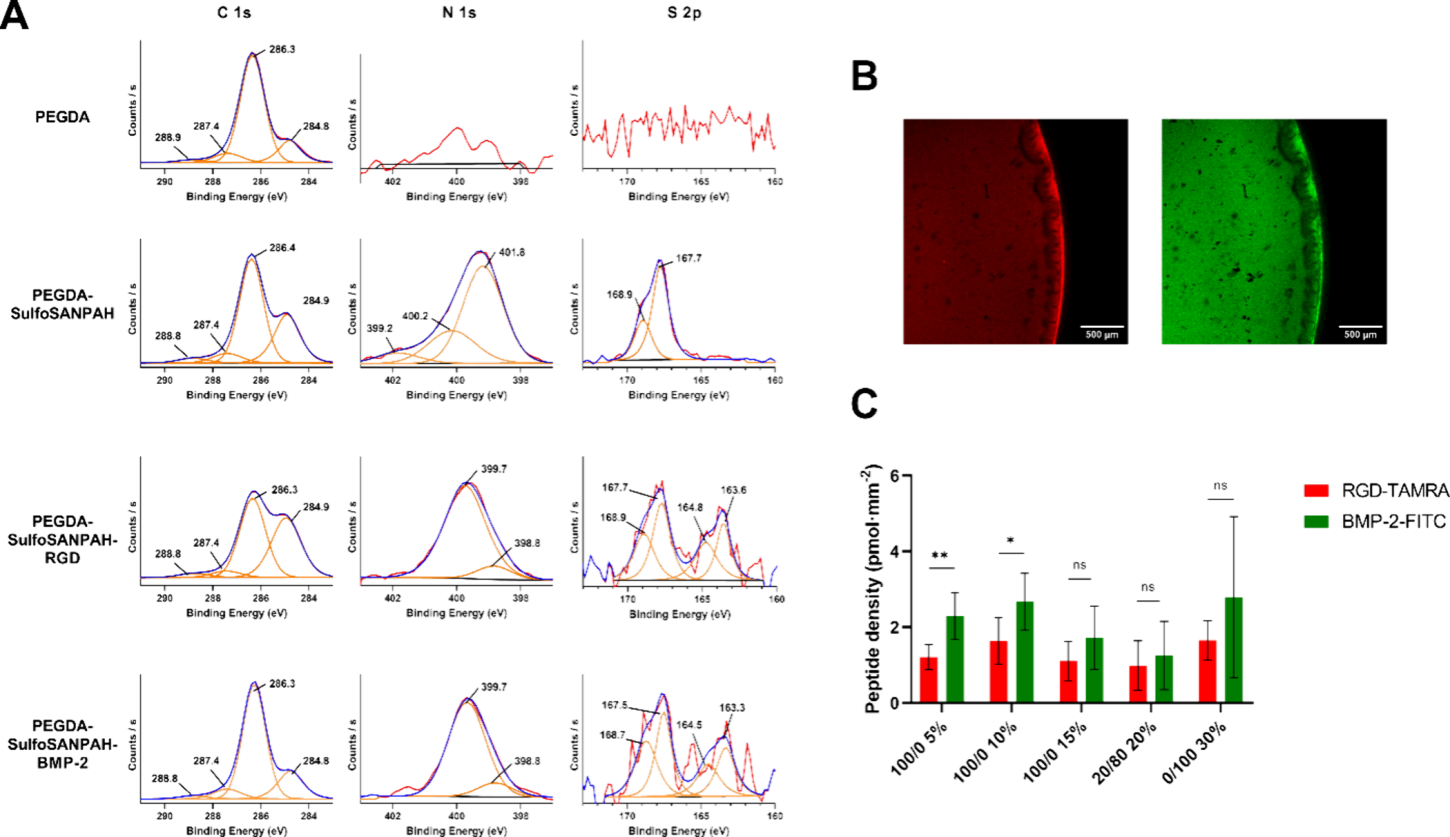
Evaluation of peptide grafting. (A) XPS high resolution spectra of carbon, nitrogen, and sulfur, of samples PEGDA, PEGDA with cross-linker SulfoSANPAH, and PEGDA with cross-linker SulfoSANPAH and either RGD or BMP-2 peptides. (B) Fluorescence microscopy images of a hydrogel treated with a combination of peptides (RGD-TAMRA and BMP-2-FITC, as detailed in the Materials and Methods section). The images correspond to the same material, under an excitation laser at 561 nm (left) or 488 nm (right). Scale bar: 500 μm (C) The surface density of RGD-TAMRA and BMP-2-FITC peptides on bifunctionalized hydrogels of various compositions following a 3-day rinsing period. The statistical analysis data are presented in the Supporting Information for clarity (Table S4).

To investigate the binding of peptides, samples of PEGDA hydrogel before and after grafting of the sulfoSANPAH cross-linker and the two peptides were analyzed by XPS. Table 1 shows the atomic composition of the surface of each sample. All samples contain the expected amounts of C and O, close to the theoretical ratio of the atoms present in the PEGDA polymer of 66% for carbon and 33% for oxygen (Table 1, Figure 4A). The high-resolution carbon spectra show two main peaks (Figure 3A). The first one at 284.8 eV is assigned to the C–C and C–H bonds, while the C–O bond corresponds to the peak at 286.3 eV. PEGDA contains a very high proportion of ether groups, which is why the peak at 286.3 eV is more prominent. The presence of nitrogen was detected in the sample only after the first step of functionalization (Figure 4A, Table 1), at 1.3 atom % in the sample grafted with the cross-linker, due to the nitrogen-containing groups present in this molecule. After the binding of the peptides, the composition of nitrogen evolved to 0.95 and 0.69 at. % for RGD and BMP-2, respectively (Table 1), and nitrogen peak clearly appears in the N 1s high-resolution spectra. The observed nitrogen environment is characteristic of protein or peptide-functionalized samples.^53,54^ Low amounts of sulfur were also detected in the grafted samples, as this atom is also present in sulfoSANPAH (Figure 4A). Sulfur is still detected in the peptide-bound samples in smaller percentages, as both peptides contain sulfur in their structures, which contributes to the XPS S 2p signal. In the sulfoSANPAH grafted material, the main peak is observed at 167.7 eV, indicating that the sulfur is in an oxidated environment. When the peptides are added, this peak remains, indicating that some sulfoSANPAH without bound peptide is still present, but a second peak of lower binding energy (∼163 eV) is also detected, which corresponds to the sulfur atoms that are bound to the carbon atom located in the cysteine amino acid.^55,56^ This clearly demonstrates the covalent linkage between the peptide and the cross-linker.

**Table 1:** Atomic Percentage of Carbon, Oxygen, Nitrogen, and Sulfur from XPS Survey Analysis of Hydrogel Materials at Each Step of the Peptide Grafting^a^

	C (%)	O (%)	N (%)	S (%)
PEGDA	68.9	30.9	0.12	
PEGDA-sulfoSANPAH	68.9	28.1	1.3	0.23
PEGDA-sulfoSANPAH-RGD	73	25	0.95	0.12
PEGDA-sulfoSANPAH-BMP-2	69.6	29.6	0.69	0.07

a Small amounts of contaminants (aluminum, silicon or calcium) were detected in some samples and are not shown here.

To further evaluate the homogeneity and density of the grafted peptides via the SulfoSANPAH cross-linker, materials functionalized with both fluorophore-coupled peptides simultaneously were studied under fluorescence microscopy. This technique has been previously used to quantify peptide density on solid surfaces^17,57^ and hydrogels.^14,58^ After grafting, the gels were thoroughly rinsed in HEPES buffer, and their fluorescence intensity was evaluated every 24 h. This rinsing process was expected to eliminate the adsorbed peptide, leading to a reduction in the fluorescence as the molecules were washed away. It was confirmed that all the adsorbed peptides were eliminated after only 1 day of vigorous agitation since the fluorescence reached a plateau after this time with no further reduction after two more days of rinsing (Figure S5). Dually functionalized materials after the rinsing process were found to exhibit green and red fluorescence and the homogeneity of the grafting of both peptides was also verified since no peptide clusters were visible (Figure 4B). By considering the fluorescence intensity at each wavelength and comparing it to the calibration curve for each peptide (Figure S6), the surface density was estimated to range from 1.0 ± 0.6 to 1.6 ± 0.4 pmol·mm^–2^ of RGD and between 1.2 ± 0.7 up to 2.8 ± 0.8 pmol·mm^–2^ of BMP-2 (Figure 4C). This means that the estimated total peptide density on the bifunctionalized materials ranges from 2.2 up to 4.4 pmol·mm^–2^ (between 1.3 and 2.6 molecules per nm^2^) depending on the hydrogel composition. The surface density of immobilized RGD peptides on the hydrogels is not significantly different among the five gel conditions. In the case of the BMP-2 peptide, only the condition 20/80 20% shows a significant difference with respect to 100/0 10 and 0/100 30% (Table S4). However, the amount of peptides is higher than the minimal threshold needed to act for osteogenic differentiation found by other researchers.^59^ By comparing the densities of immobilized RGD and BMP-2 peptides on each hydrogel condition, in all cases, more of the osteogenic peptide was conjugated, although the difference was only significant in the case of the samples 100/0 5 and 100/0 10%. These values are similar to those already reported for other hydrogels^14,60^ and solid surfaces,^17,57,61^ although slightly higher in many cases. This could be explained because the peptide is grafted not only on the extreme surface but also can be found in the bulk of the hydrogel due to diffusion during the conjugation process. Even though the technology of confocal microscopy enables us to eliminate the out-of-plane fluorescence, since the images are acquired with an objective at low magnification, the resolution in *Z* is relatively large (25–30 μm depending on the laser wavelength), and therefore, some of the captured fluorescence will be due to peptides present in layers below the surface.

### Effects of Hydrogel Mechanical and Bioactive Properties on Osteogenic Differentiation

As a reminder, the objective of this work was to assess the impact of the mechanical properties of hydrogels (elasticity and viscoelasticity) on the commitment of mesenchymal stem cells toward an osteogenic lineage. All hydrogels were prefunctionalized with RGD and BMP-2 peptides to promote not only the adhesion of hMSCs but also their differentiation toward an osteogenic lineage. Three out of the five hydrogels (100/0 5, 100/0 15, 0/100 30%) were selected, with shear storage moduli of 1, 10, and 42 kPa and half-time relaxations of 1963, 434, and 60 s, respectively, to cover a wide range of elastic and viscoelastic properties. Cell adhesion and spreading were evaluated after 24 h, and the osteogenic differentiation was evaluated through immunocytochemistry after 2 weeks of culture in basal medium without osteogenic factors and after 1 week in osteogenic medium. This technique allows quantification of the expression of different protein markers and provides information about the cell shape and organization.

It is observed that PEGDA functionalized with the combination of RGD and BMP-2 peptides facilitates cell adhesion at 24 h without serum and throughout the 1- and 2-week experiments. This contrasts with the case of virgin PEGDA, where no adhesion is possible (data not shown). After 24 h on the materials, the cell area was found to be higher in the stiffest substrate, compared to the two others with soft and medium stiffness (Figure S7), as observed in previous studies.^11,62^ In addition, cells on the medium and stiff substrates present highly organized actin fibers (Figure S7).

**Figure 5:**
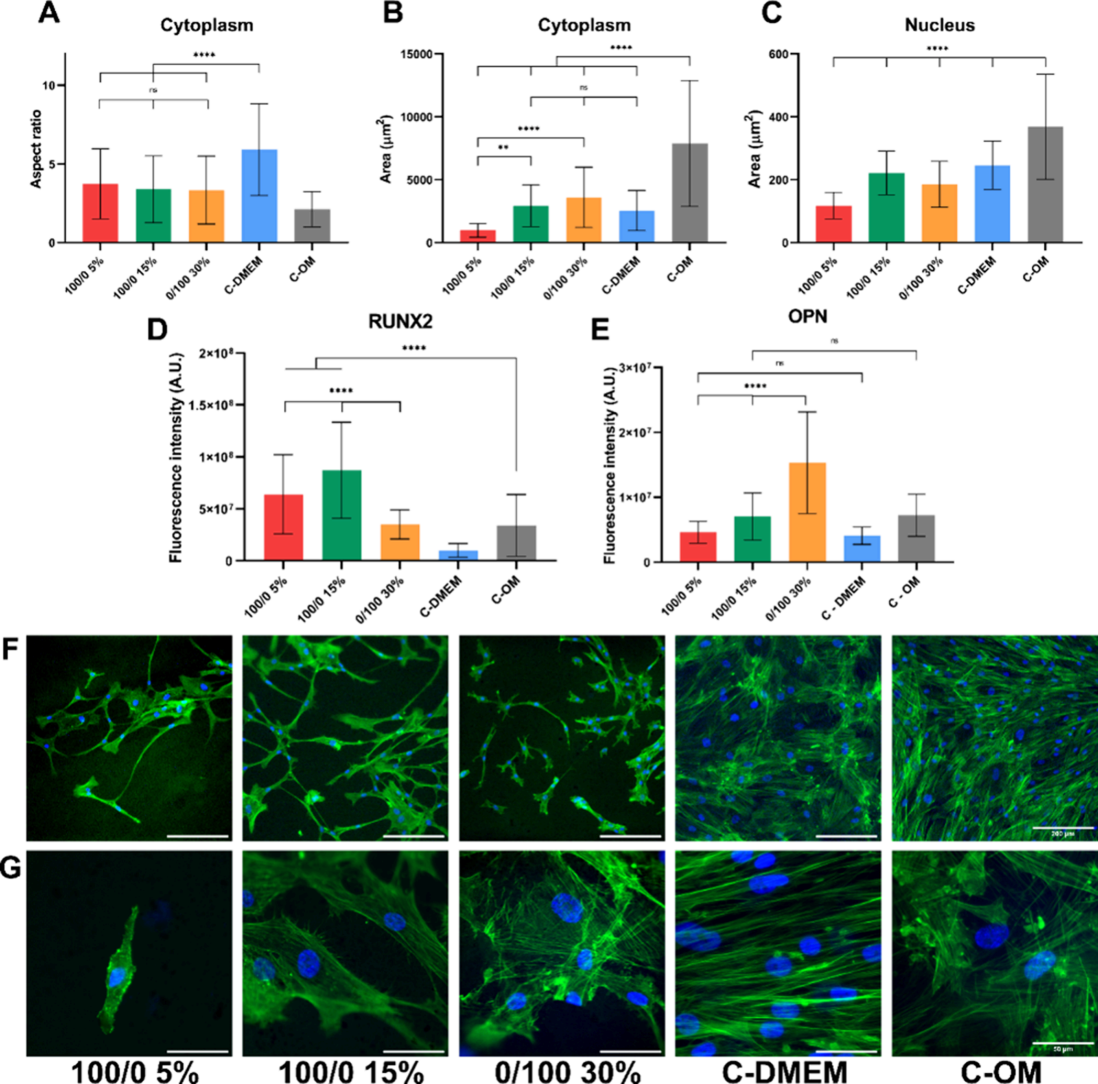
Evaluation of osteogenic differentiation after 2 weeks in basal medium. (A–C) Morphological evaluation of cells on hydrogel substrates and controls. (A) Quantification of cellular aspect ratio. (B) Measurement of total cell area. (C) Measurement of nuclear area. (D) Quantitative analysis of the expression of Runx2. (E) Quantitative analysis of the expression of OPN. (F,G) Representative immunofluorescence images of cells on the different hydrogels and controls with the cytoskeleton (green) and nucleus (blue). (F) Scale bar = 200 μm. (G) Scale bar = 50 μm.

After 2 weeks of culture, the distribution and morphology of the cells were assessed by visualizing the cell cytoskeleton stained with phalloidin. To provide a quantitative evaluation of cell shape, the aspect ratio (AR) of cells cultured on all materials and controls was evaluated (Figure 5A). An aspect ratio of 1 corresponds to a perfect circle or square, while higher values indicate a more elongated shape. Following a 2-week culture period, the cell morphology in the control group 'C-DMEM' retained its elongated and flattened shape with an AR value of around 6, which is a characteristic hallmark of hMSCs (Figure 5A, F).^63^ Conversely, the cells observed on the 'C-OM' control materials exhibited a notable morphological transformation after 2 weeks of culture, adopting a cuboidal shape indicative of osteoblastic differentiation,^18^ with the lowest AR among all the conditions (around 2) (Figure 5A, F). Cells on the softer material (100/0 5%) were smaller and less spread, while cells on the other two hydrogels (100/0 15 and 0/100 30%) appeared to be larger and with more defined actin fibers. Some cells with cuboidal morphology can be observed on medium- and high-stretch hydrogels, which is the typical morphology of osteoblasts. The aspect ratios of cells cultured under the three hydrogel conditions exhibited intermediate values, ranging between 3 and 4. Although the cell aspect ratios among the three hydrogel conditions are not significantly different (Figure 5A), the aspect ratios of the hydrogels are significantly different from the control group in DMEM. This highlights quantitative differences in the cell shape. The AR should be carefully considered in conjunction with qualitative observations as well as the total cell spread area. Indeed, the cells on the 100/0 5% material have similar AR to the other hydrogels but a considerably lower area (Figure 5B), indicating that they are small and round, similar to what other studies have found on soft materials that do not favor osteogenic differentiation.^14,62,64^ Clear differences in the nuclear area are observable between the control groups “C-DMEM” and “C-OM,” with the latter being around 1.5 times larger than the former (Figure 5C). Indeed, YAP nuclear translocation, which is a key mechanotransduction pathway involved in osteogenic differentiation, has been correlated with increases in nuclear sizes.^65,66^ The hydrogels with medium and high stiffness have a nuclear area similar to that of “C-DMEM”, while cells on the softest material have significantly smaller nuclei (Figure 5C).

To investigate cell differentiation toward the osteogenic lineage, the expression of the early osteogenic marker Runx-2 and the osteoblast marker osteopontin (OPN) were evaluated after 2 weeks of culture (Figure 5D, E). It is important to emphasize that depending on the hydrogel's chemical, biochemical, and mechanical properties, not all hMSCs differentiate with the same differentiation kinetics. Therefore, after 2 weeks, the cells are not all at the same differentiation stage, which calls for the need to assess more than one differentiation marker to understand the progression of osteogenesis. Interestingly, our results highlight a significant overexpression of Runx-2 for all three hydrogels in comparison with the two controls “C-DMEM” and “C-OM,” which indicates the commitment of cells toward osteoblastic differentiation. The hydrogel that shows the highest expression of Runx-2 is 100/0 15%, which has an elastic modulus of 50 kPa and a relaxation around 50% measured in compression. In contrast, the stiffest hydrogel (*E* = 128 kPa) exhibited the lowest expression of this protein, while, at the same time, it had the highest expression of OPN. This is likely an indicator that cells on the 0/100 30% are more advanced in the osteogenic differentiation process since OPN is a late osteoblastic marker and overexpression of Runx-2 can inhibit osteoblast maturation.^67^ This differentiation toward the osteogenic lineage is achieved solely from the combined action of the mechanical and biological properties of the hydrogel substrate, without the addition of any differentiation factors in the culture medium.

The differences among materials indicate that not only the peptide functionalization is acting to induce differentiation but there is also a role of the substrate mechanical properties. These observations are consistent with previous studies that showed that biochemical and mechanical cues act together to modify cell behavior. For instance, Blackford et al.^68^ demonstrated that changing the concentration of an RGD peptide on a PEG-based matrix had opposing effects on hepatocyte cells depending on the stiffness of the material. This highlights the importance of controlling each of these variables independently. Specifically for MSCs, Li et al.^69^ showed elevated activity of alkaline phosphatase and expression of osteogenic genes for MSCs that were cultured with osteogenic medium on stiff materials (elastic modulus of 74 kPa) grafted with a BMP-2 mimetic peptide.

**Figure 6:**
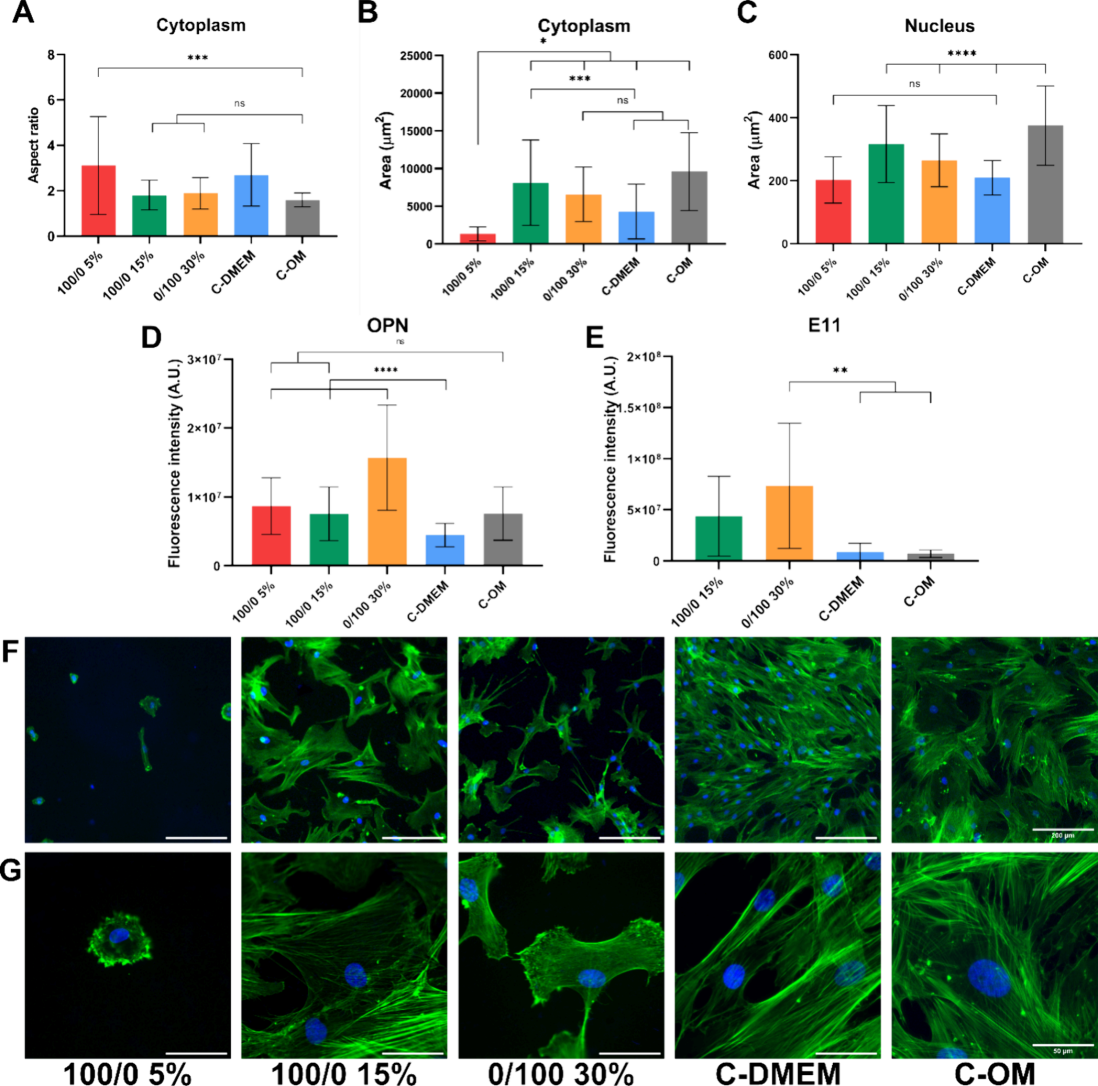
Evaluation of osteogenic differentiation after 1 weeks in induction medium. (A–C) Morphological evaluation of cells on hydrogel substrates and controls. (A) Quantification of cellular aspect ratio. (B) Measurement of total cell area. (C) Measurement of nuclear area. (D) Quantitative analysis of the expression of OPN. (E) Quantitative analysis of the expression of E11. (F,G) Representative immunofluorescence images of cells on the different hydrogels and controls with cytoskeleton (green) and nucleus (blue). (F) Scale bar = 200 μm. (G) Scale bar = 50 μm.

The rapid obtention of committed cells is of interest for clinical tissue engineering applications, and the synergistic effects of soluble factors with material properties offer a promising strategy to achieve this. To explore this, cell differentiation toward the osteogenic lineage was further investigated in the presence of induction culture media after only 1 week of culture. Regarding cell shape descriptors, the AR of cells on materials 100/0 15 and 0/100 30% decreased with respect to the experiment in basal medium, which confirms the qualitative observation that cells present a cuboidal morphology (Figure 6A, F, G). Specifically, the cells on the 100/0 15% material exhibited large polygonal shapes resembling those in the control group ‘C-OM,’ while cells on the 0/100 30% material appeared smaller with multiple dendritic processes that might be indicative of osteocyte commitment. The softest samples have an AR that presents a very high standard deviation since cells on these materials present varied morphologies that go from elongated to small and round. As in the previous experiment in basal media, cells in this condition exhibit the smallest cytoplasmic area (Figure 6B). As for the nucleus size, no significant differences were found between those on the soft sample and the control group “C-DMEM.” On the contrary, those on the two other hydrogel conditions present enlarged nuclei, being the largest for those on the sample 100/0 15%, which also present the largest total cell spread area.

The markers chosen to evaluate these conditions are OPN and podoplanin (E11), which is an early osteocyte marker (Figure 6D, E). As observed previously, all the hydrogel conditions present an overexpression of OPN as compared to the controls on glass. The expression of OPN on the sample 100/0 15% matched that of the control group “C-OM” and doubled in cells on the stiffest material (0/100 30%). To evaluate the potential commitment of cells to osteocyte formation, these two conditions were stained with E11. Expression of E11 was minimal in the controls and did not significantly differ between them regardless of the presence of a differentiation medium. Nevertheless, it was boosted on the two materials and expressed at the highest level in the samples 0/100 30%, confirming the conclusion of the morphological observations that cells on this condition display osteocyte-like characteristics. The obtention of osteocytes in vitro is rare and generally reported in three-dimensional cultures. The material 0/100 30%, which appears to favor osteocyte-like cell formation, has a shear modulus of 40 kPa in rheology (*E* = 150 kPa under macroscopic compression), which is higher than what is generally reported to favor osteogenic differentiation.^8,9^ However, this material is also viscoelastic, presenting the fastest relaxation when tested under rheology. Taking into consideration the results obtained regarding aspect ratio, cell area, nucleus area, and OPN and E11 expression, we can affirm that two hydrogel conditions, 100/0 15 and 0/100 30%, allow us to either mimic our positive control, which is glass with osteogenic medium (C-OM), or surpass it in terms of osteogenic differentiation. Overall, the 0/100 30% condition leads to the most advanced differentiation, showing the highest overexpression of E11 and OPN, both indicative of osteocyte differentiation.^70^

Our study highlights for the first time that evaluating the mechanical properties (elasticity and viscoelasticity) of hydrogels using various techniques across different scales reveals a consistent trend, even though the measured values may differ. The precise elastic and viscoelastic properties conducive to osteogenesis are yet to be established, despite numerous studies focusing on the impact of elasticity on MSC differentiation. A few studies offer an evaluation not only of elasticity but also of viscoelasticity. Some authors have suggested that higher viscoelasticity in hydrogels could enhance osteogenic differentiation,^13,14,38,62^ in studies in which different materials, culture conditions, and elastic moduli are used. Our hypothesis, in conclusion, is that the fast-relaxation properties of the hydrogels can offset the nonoptimal rigidity and ultimately promote the progression of osteogenic differentiation.

## Conclusions

In this study, we fabricated biofunctionalized PEGDA hydrogels and conducted a comprehensive mechanical characterization, demonstrating their ability to support MSC adhesion and proliferation and, depending on their properties, enhance osteogenic differentiation.

In summary, our work shows that characterization of the rigidity of hydrogels with compression, rheology, and AFM leads to results that are similar in tendency but differ in terms of the absolute values that are measured, emphasizing the need for caution when comparing data independently obtained in the literature through different techniques. Notably, our investigation confirms that the surface properties of PEGDA hydrogels are in line with the ones measured in the bulk, and thus, macroscopic techniques such as rheology or compression are sufficient to characterize these materials for cell culture applications. We also showed that the most common techniques to characterize viscoelasticity, including stress-relaxation in compression, rheology, and AFM, and dynamic testing in rheology and AFM, provide results that are very similar in tendency. This is the first instance, to the best of our knowledge, in which a cross-technique viscoelastic characterization of hydrogels has been discussed in the literature, and given the rising popularity of studies regarding the effect of viscoelastic materials, we believe it is an important factor to account for.

Finally, we also demonstrate that the mechanical properties of these PEGDA hydrogels act synergistically with the presence of RGD and BMP-2 peptides to favor the osteogenic differentiation of MSCs. Overall, by examining the hydrogel chemistry, evaluating the effectiveness of the functionalization, and characterizing the mechanical properties in terms of elasticity and stress relaxation, this research provides the groundwork for future studies aiming to unlock the full potential of PEGDA hydrogels in facilitating controlled and reproducible hMSC differentiation, ultimately enhancing the prospects for successful clinical applications.

## Supporting Information Available

Hydrogel reaction scheme, evolution of hydrogel swelling, strain sweep, AFM microrheology, evolution of fluorescence during rinsing period, calibration curves of RGD and BMP-2, cell adhesion after 24 h, and statistical analyses (PDF)
