# Supplementary material for: Integrating Mechanics and Bioactivity: A Detailed Assessment of Elasticity and Viscoelasticity at Different Scales in 2D Biofunctionalized PEGDA Hydrogels for Targeted Bone Regeneration
Source: ACS Appl Mater Interfaces. 2024 Jul 23;16(30):39165–80. doi: 10.1021/acsami.4c10755 (PMC11600396; doi:10.1021/acsami.4c10755)
Supplement: Supplementary file 1 — am4c10755_si_001.pdf [file am4c10755_si_001.pdf]

Supporting Information

Integrating Mechanics and Bioactivity: A Detailed Assessment of Elasticity and Viscoelasticity at Different Scales in 2D Biofunctionalized PEGDA Hydrogels for Targeted Bone Regeneration

Cristina López-Serrano,[1,2,3] Yeva Côté-Paradis,[1,2] Birgit Habenstein,[4] Antoine Loquet,[4] Cédric Le Coz,[5] Jean Ruel,[6] Gaétan Laroche,[1,2][#] Marie-Christine Durrieu[3]*[#]*

[1]Laboratoire d'Ingénierie de Surface, Centre de Recherche sur les Matériaux Avancés, Département de Génie des Mines, de la Métallurgie et des Matériaux, Université Laval, Québec, Canada.

[2]Axe médecine régénératrice, Centre de Recherche du Centre Hospitalier Universitaire de Québec, Hôpital St-François d'Assise, Québec, Canada

[3]Univ. Bordeaux, CNRS, Bordeaux INP, CBMN, UMR 5248, Pessac, France

[4] Univ. Bordeaux, CNRS, INSERM, IECB, US1, UAR 3033, F-33600 Pessac, France

[5]Univ. Bordeaux, CNRS, Bordeaux INP, LCPO, UMR 5629, Pessac, France

[6]Département de Génie Mécanique, Université Laval, Québec, Canada.

[#]These authors equally contributed

*Corresponding authors

marie-christine.durrieu@inserm.fr

Gaetan.Laroche@gmn.ulaval.ca

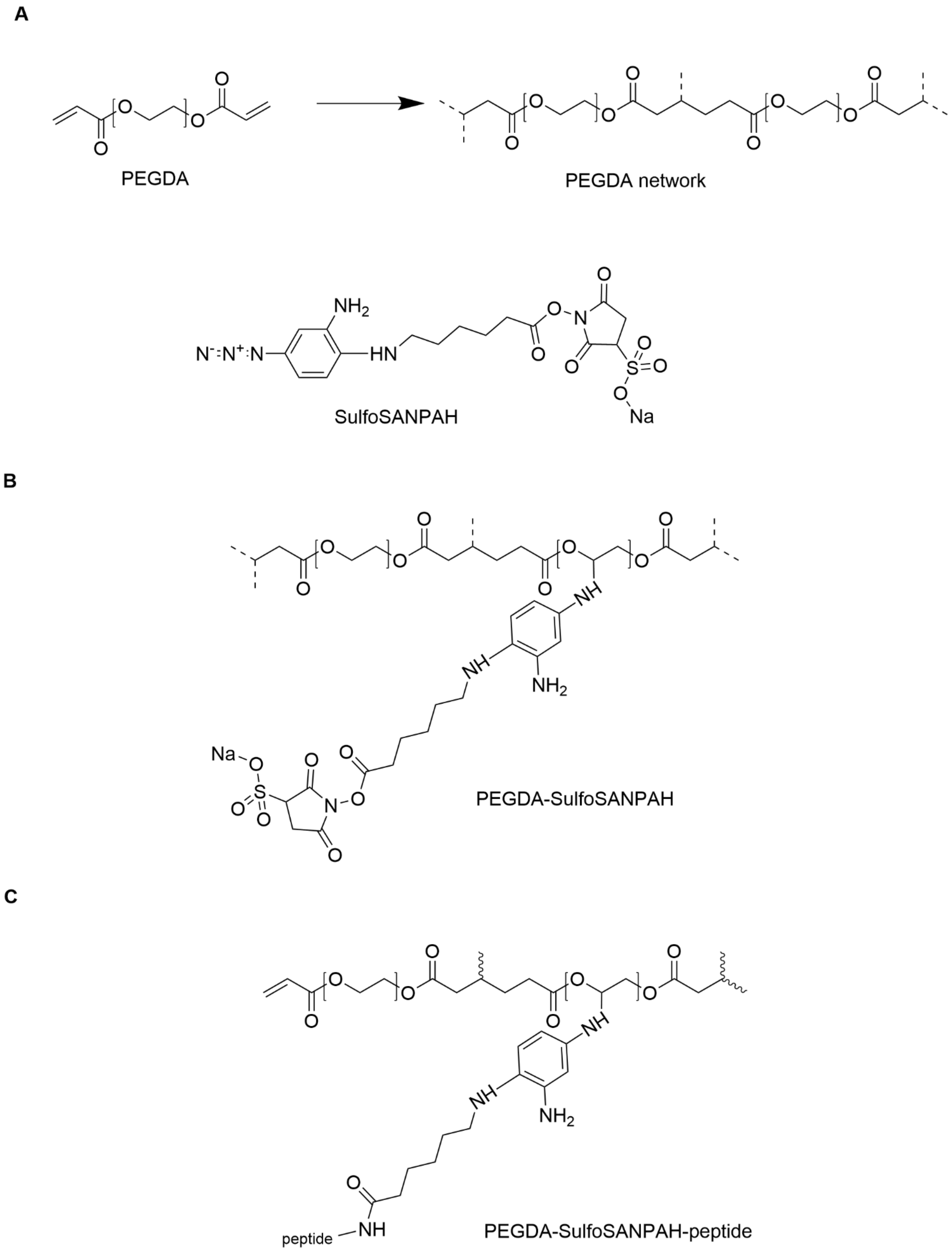

Figure S1: Reaction scheme showing the formation of the PEGDA hydrogel (a) and the functionalization process (b and c).

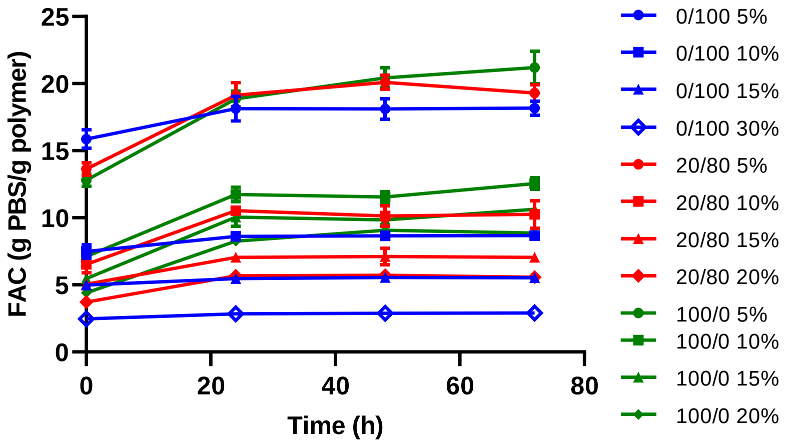

Figure S2: Evolution of hydrogel swelling from synthesis (t=0) up to 3 days swelling in PBS at 4ºC. The hydrogels are fully swollen after 24h.

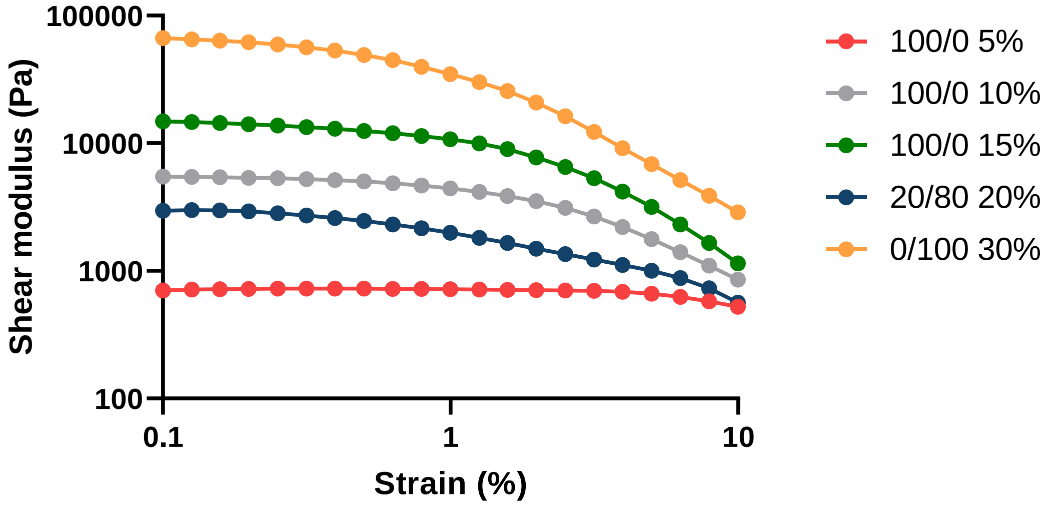

Figure S3: Strain sweep performed at 1Hz for the different hydrogel compositions to determine the linear viscoelastic range.

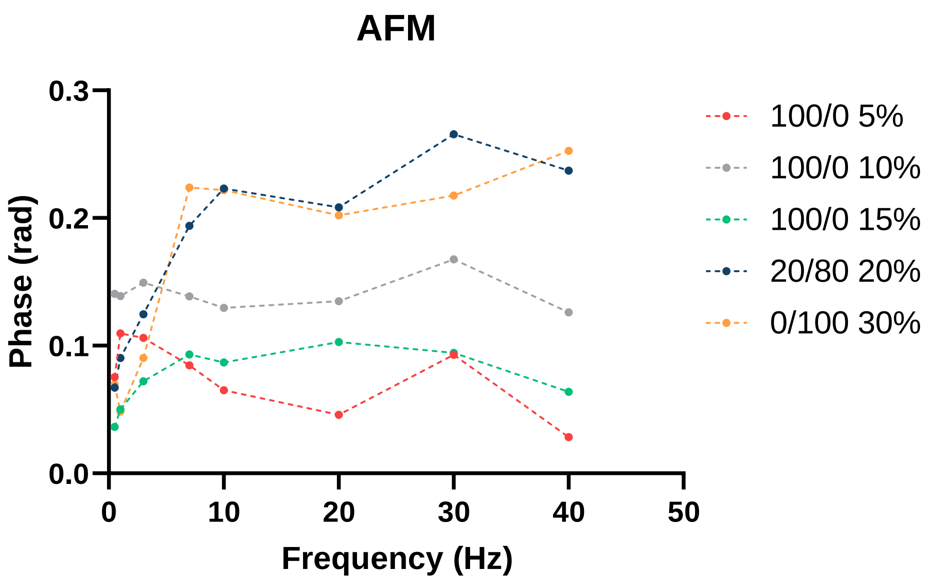

Figure S4: AFM microrheology testing of 5 different hydrogels at frequencies from 0.5 to 40Hz.

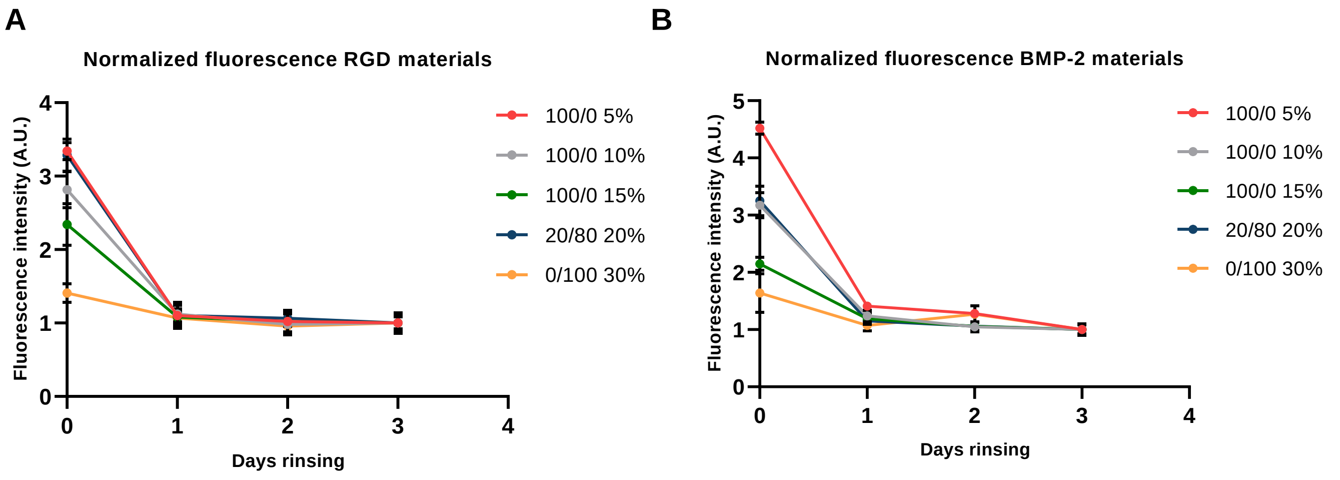

Figure S5: Normalized fluorescence intensity of a) hydrogels functionalized with a fluorescently labelled RGD peptide or b) BMP-2 peptide, as a function of the number of days of rinsing, normalized to the value on the third day. The fluorescence intensity is stable after 1 day of rinsing and remains stable up to at least 3 days of rinsing.

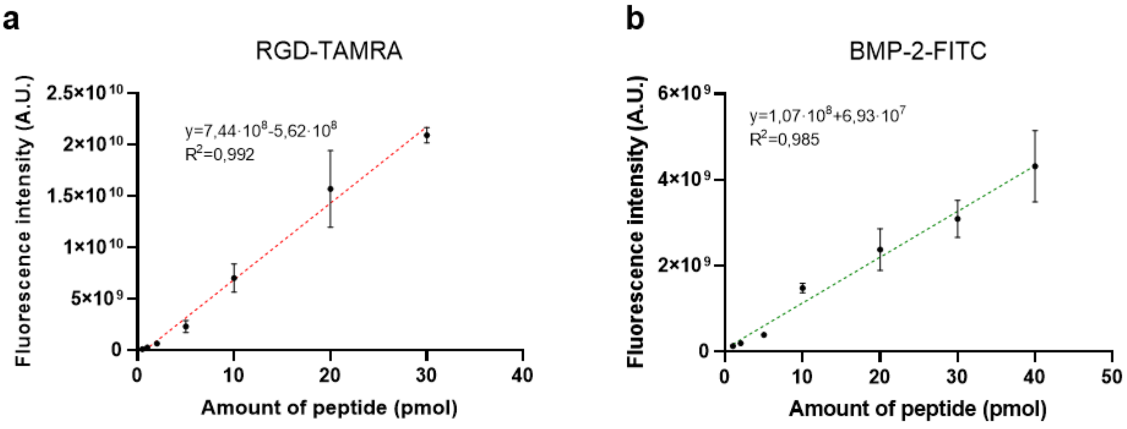

Figure S6: Fluorescence standard curves of RGD-TAMRA and BMP-2-FITC peptides as a function of the total amount of peptide in picomoles.

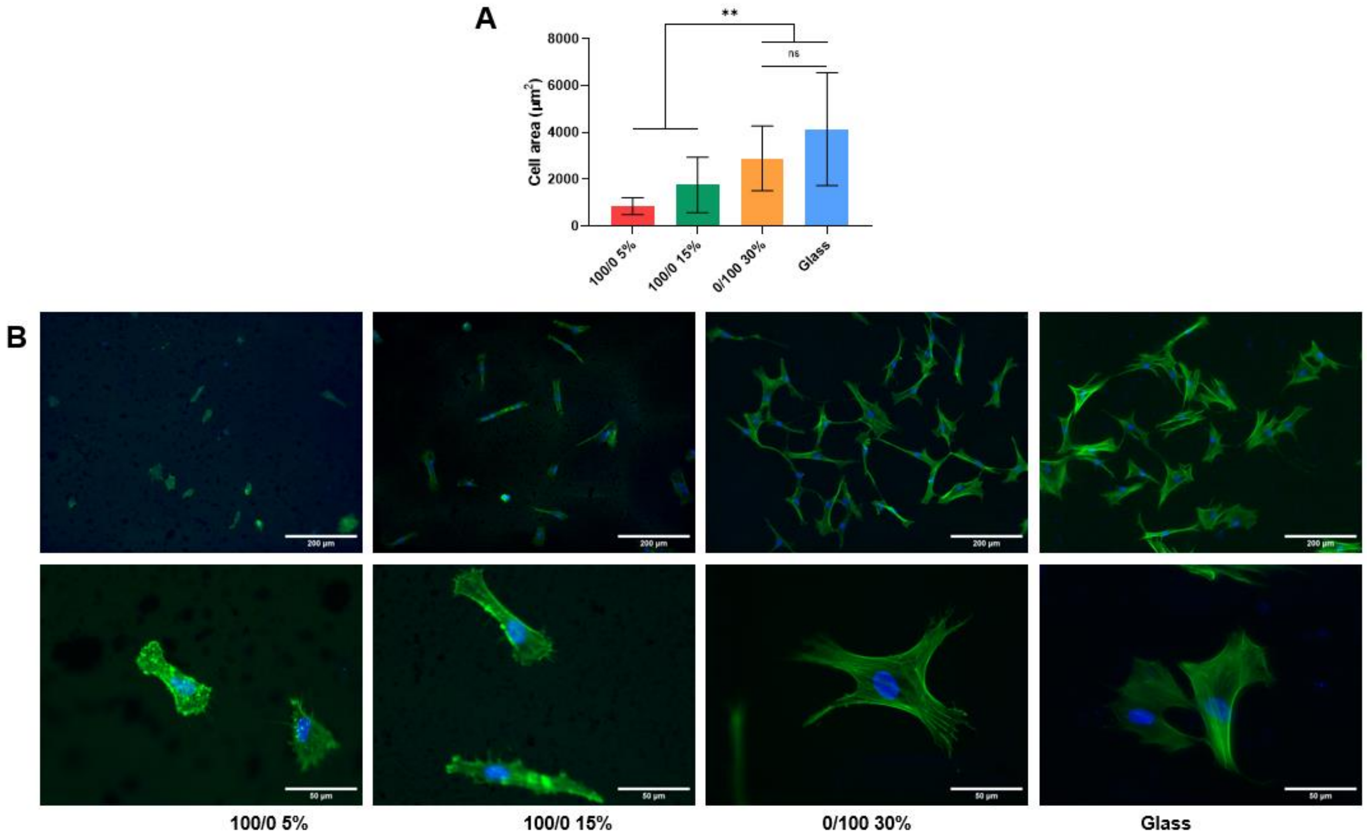

Figure S7: (A) Analysis of cell spread area after 24 hours. (B) Representative immunofluorescence images of each condition. Scale bar = 200 µm (top) and 50 µm (bottom).

	0/100 5%	0/100 10%	0/100 15%	0/100 20%	0/100 30%	20/80 5%	20/80 10%	20/80 15%	20/80 20%	100/0 5%	100/0 10%	100/0 15%	100/0 20%
0/100 5%		****	****	****	****	****	****	****	****	****	****	****	****
0/100 10%			****	****	****	****	**	*	****	****	****	****	ns
0/100 15%				****	****	****	****	**	ns	****	****	****	****
0/100 20%					ns	****	****	****	****	****	****	****	****
0/100 30%						****	****	****	****	****	****	****	****
20/80 5%							****	****	****	ns	****	****	****
20/80 10%								****	****	****	****	ns	*
20/80 15%									*	****	****	****	**
20/80 20%										****	****	****	****
100/0 5%											****	****	****
100/0 10%												***	****
100/0 15%													***
100/0 20%													

Table S1: Statistical analyses of hydrogel fluid absorption capacity (FAC). Statistical analyses were done by one-way analysis of variance (ANOVA) and Tukey's test for multiple comparisons. P values are represented as following * ≤ 0.05, ** ≤ 0.01, *** ≤ 0.001, **** ≤ 0.0001. ns indicates absence of statistical significance at alpha equal to 0.05.

**(A)** Compression Young's Modulus	100/0 5%	100/0 10%	100/0 15%	20/80 20%	0/100 30%
100/0 5%		ns	*	ns	*
100/0 10%			*	ns	*
100/0 15%				ns	ns
20/80 20%					*
0/100 30%					

**(B)** Rheology Storage Modulus	100/0 5%	100/0 10%	100/0 15%	20/80 20%	0/100 30%
100/0 5%		****	***	**	*
100/0 10%			**	***	*
100/0 15%				***	*
20/80 20%					*
0/100 30%					

**(C)** AFM Young's Modulus	100/0 5%	100/0 10%	100/0 15%	20/80 20%	0/100 30%
100/0 5%		ns	****	****	****
100/0 10%			****	****	
100/0 15%				****	ns
20/80 20%					****
0/100 30%					

Table S2: Statistical analysis of elasticity measurements: (A) Compression Young's modulus, (B) Rheology storage modulus, (C) AFM Young's modulus.

**(A)** Compression stress-relaxation	100/0 5%	100/0 10%	100/0 15%	20/80 20%	0/100 30%
100/0 5%		ns	*	ns	ns
100/0 10%			ns	ns	ns
100/0 15%				*	ns
20/80 20%					ns
0/100 30%					

**(B)** AFM stress-relaxation	100/0 5%	100/0 10%	100/0 15%	20/80 20%	0/100 30%
100/0 5%		***	****	****	****
100/0 10%			****	ns	****
100/0 15%				**	ns
20/80 20%					****
0/100 30%					

**(C)** Rheology stress-relaxation	100/0 5%	100/0 10%	100/0 15%	20/80 20%	0/100 30%
100/0 5%		**	**	**	**
100/0 10%			ns	ns	*
100/0 15%				ns	*
20/80 20%					*
0/100 30%					

**(D)** Rheology loss tangent	100/0 5%	100/0 10%	100/0 15%	20/80 20%	0/100 30%
100/0 5%		ns	***	****	*
100/0 10%			**	***	*
100/0 15%				***	ns
20/80 20%					ns
0/100 30%					

(E) AFM loss tangent	100/0 5%	100/0 10%	100/0 15%	20/80 20%	0/100 30%
100/0 5%		****	ns	****	****
100/0 10%			ns	*	*
100/0 15%				****	****
20/80 20%					ns
0/100 30%					

Table S3 Statistical analysis of viscoelasticity measurements: (A) Compression stress-relaxation, (B) AFM stress-relaxation, (C) Rheology stress-relaxation, (D) Rheology loss tangent, (E) AFM loss tangent.

a RGD-TAMRA

	100/0 5%	100/0 10%	100/0 15%	20/80 20%	0/100 30%
100/0 5%		ns	ns	ns	ns
100/0 10%			ns	ns	ns
100/0 15%				ns	ns
20/80 20%					ns
0/100 30%					

b BMP-2-FITC

	100/0 5%	100/0 10%	100/0 15%	20/80 20%	0/100 30%
100/0 5%		ns	ns	ns	ns
100/0 10%			ns	*	ns
100/0 15%				ns	ns
20/80 20%					*
0/100 30%					

Table S4: Statistical analyses functionalization quantification.
